# Floral Color and Family Drive Contrasting Plant–Pollinator Responses to Nutrient Enrichment

**DOI:** 10.1002/ece3.72153

**Published:** 2025-09-13

**Authors:** Rebecca A. Nelson, Elizabeth T. Borer, Eric W. Seabloom

**Affiliations:** ^1^ Department of Wildland Resources and the Ecology Center Utah State University Logan Utah USA; ^2^ Department of Environmental Science & Policy University of California, Davis Davis California USA; ^3^ Department of Integrative Biology Michigan State University East Lansing Michigan USA; ^4^ Department of Ecology, Evolution, and Behavior University of Minnesota St. Paul Minnesota USA

**Keywords:** eutrophication, functional traits, global change ecology, grasslands, nutrient network, plant–pollinator interactions, species interaction networks

## Abstract

Nutrient enrichment has decreased the diversity and abundance of wildflower species, raising questions about whether nutrient enrichment can further decrease the diversity and abundance of pollinators that rely on wildflowers. Whether the effects of nutrient enrichment on plant–pollinator interactions differ by nutrient type remains an open question. Moreover, plant family and flower color, two core axes of pollination niches, may further mediate how wildflowers and their pollinators respond to nutrient enrichment. We tested these questions using a nutrient addition experiment replicated at three grasslands in California, a global plant diversity hotspot. We found that adding nitrogen increased the floral abundance of Asteraceae, while decreasing that of Fabaceae, Geraniaceae, Iridaceae, and Euphorbiaceae. Adding phosphorus and potassium in the absence of nitrogen produced the opposite effects. Pollinator abundance and composition varied strongly by floral family, suggesting that these differing responses to nutrient addition by floral family may alter pollinator community composition. Nitrogen addition decreased the abundance of native blue, native green, and exotic pink flowers, while increasing the abundance of native and exotic yellow and exotic purple flowers. Consequently, nitrogen addition increased pollinator abundance on purple flowers, while decreasing pollinator abundance on pink flowers. Purple and yellow Asteraceae species, which increased under nitrogen enrichment, acted as core hubs in structuring the plant–pollinator network. *Synthesis:* Our findings suggest that the type of nutrient, plant family, and flower color modulate how plant–pollinator interactions respond to eutrophication.

## Graphical abstract

Floral taxonomic family and color mediated how plant–pollinator reactions respond to nutrient enrichment. Nitrogen enrichment increased yellow and purple Asteraceae flowers and decreased pink, blue, and green flowers in Geraniaceae, Fabaceae, and Iridaceae. These shifts in floral colors and traits altered pollinator composition.

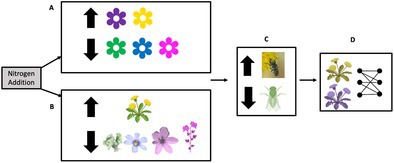

## 1 Introduction

Forbs (non‐graminoid, herbaceous wildflowers) contribute to grassland functional diversity, providing essential food resources to pollinators (Ollerton et al. 2011; Hallett et al. 2017; Maestre et al. 2022). However, forbs are declining in diversity and abundance due to increasing competition with grasses under anthropogenic nutrient enrichment (Song et al. 2011; Valliere et al. 2020; Nelson et al. 2025). Nutrient enrichment can produce functional shifts in forb community assembly and composition (Midolo et al. 2019; Wilcots et al. 2021; Garbowski et al. 2023). Decreases in forb diversity and abundance under nutrient enrichment can lead to declines in pollinator abundance and diversity (Veen et al. 2024). Nutrient enrichment can either decrease or enhance the quality of floral rewards forbs provide to pollinators through altering nectar and pollen chemistry (Gijbels et al. 2015; Ceulemans et al. 2017; David et al. 2019) and shifting flowering phenology to earlier in the season (Biederman et al. 2017). Moreover, through changing forb community composition, nutrient enrichment can produce functional shifts in pollinator composition (Carvalheiro et al. 2020; Dyer et al. 2021). Most of these prior findings have focused on nitrogen addition and on the aggregate response of all forb species (David et al. 2019), raising questions about whether the effects of nutrient enrichment on plant–pollinator mutualisms differ by nutrient type and by floral traits.

Whether plant–pollinator responses to nutrient addition differ by type and number of nutrients added remains an open question, yet considering multiple nutrients is more reflective of real‐world scenarios of global change (Fay et al. 2015). While nitrogen addition, in particular, has driven declines in forb diversity and abundance (Song et al. 2011; Valliere et al. 2020; Nelson et al. 2025), the addition of nitrogen, phosphorus, and potassium with micronutrients together can have synergistic and additive effects on grassland productivity (Fay et al. 2015) and forb community composition (Wilcots et al. 2021), suggesting that subsequent effects on pollinators may vary by type and number of nutrients. Variation in floral traits may further mediate differences in forb response by nutrient type (Carvalheiro et al. 2020; Tognetti et al. 2021). Whether plant traits can explain the effects of global change on plant–pollinator interactions remains an ongoing debate in ecology (Blonder et al. 2014; Rosas‐Guerrero et al. 2014; Funk et al. 2017; Valdovinos and Marsland 2021).

Flower color and plant family identity are two important axes of a plant's pollination niche (Dellinger 2020; Phillips et al. 2020). Many pollinators will specialize in specific families of forbs (Thorp and Leong 1998), as species within floral families often have similar flower morphologies and nutrient acquisition strategies (Baldwin and Goldman 2012; Rosas‐Guerrero et al. 2014). Flower color, which can vary considerably within a plant family, can explain the structure of plant–pollinator interactions (LeCroy et al. 2021; Albor et al. 2022) and further distinguish between pollination niches or “syndromes” as bees often visit blue and yellow flowers, birds red flowers, flies green and orange flowers, and moths white flowers (Rosas‐Guerrero et al. 2014; Dellinger 2020) [but see (Reverté et al. 2016)]. These pollination niches may reflect coevolution of pollinator groups and flower colors (Rausher 2008; Kellenberger and Glover 2023). Competition for pollinators among plant species may have driven the evolution of complementary and diverse flower colors among co‐flowering plant communities (Shrestha et al. 2019). Across insect taxa, pollinators vary in color perception, but share some perceptions of color with vertebrates (Song and Lee 2018; Garcia et al. 2020).

Common forb families differ in their responses to nutrient addition (Tognetti et al. 2021; Nelson et al. 2025) in ways that may have functional consequences for pollinators. While nitrogen addition decreased forb diversity and abundance across a broad range of forb families (especially Asteraceae and Fabaceae) (Valliere et al. 2020; Nelson et al. 2025), phosphorus and potassium in the absence of nitrogen can increase the diversity and abundance of nitrogen‐fixing legumes in Fabaceae (Tognetti et al. 2021). Further evidence suggests that potassium addition can decrease the abundance of invasive asters within Asteraceae (Tilman et al. 1999). These differing responses of forb families by type of nutrient may shift the composition of pollinator assemblages (Carvalheiro et al. 2020). Members of Asteraceae tend to have flat, composite, later‐season flowers accessible to a wide variety of pollinators, while members of Fabaceae tend to have deeper, early‐season flowers accessible to long‐tongued pollinators (Mciver et al. 2009; Gibson et al. 2019; Russo et al. 2019).

Flower colors may further differ in their responses to nutrient addition. Blue flowers, which are rarer in grasslands and often bee‐pollinated, are declining under nutrient addition (Dyer et al. 2021) [but see (Apland et al. 2025]). More common flower colors in animal‐pollinated systems like purple and yellow, which also tend to be bee‐pollinated, may be less sensitive to nutrient enrichment (Dyer et al. 2021). Less common flower colors like pink, which tend to be bee‐pollinated, and green, which tends to be fly‐pollinated, may similarly decline under nutrient enrichment like blue flowers. Moreover, invasive forbs, which tend to increase under nutrient addition in contrast to native forbs (Seabloom et al. 2009), often differ in their flower color spectra from those of native forbs, with invasive forbs having fewer blues (Sooraj et al. 2019). Shifts within the color composition of forb communities under nutrient addition may lead to differences in response across pollinator functional groups that vary in color preferences (e.g., bees, flies) (Carvalheiro et al. 2020; Dyer et al. 2021). Declines in blue and green flowers under nitrogen addition may decrease flies and long‐tongued bee species that may specialize in these colors (Carvalheiro et al. 2020, Dyer et al. 2021).

Trait‐matching can influence the structure and stability of plant–pollinator networks (Schleuning et al. 2015; Valdovinos et al. 2018; Peralta et al. 2020; Lautenschleger et al. 2021) as can shifts in plant abundances (Bartomeus et al. 2008; Russo et al. 2019; Parra‐Tabla and Arceo‐Gómez 2021). Thus, changes arising from nutrient enrichment in the relative abundance of flowers with differing traits can have subsequent effects on plant–pollinator network structure (Wang et al. 2022). Nutrient enrichment may alter which plant species act as functionally significant, centralized hubs for pollinators within the network that strongly influence network structure by attracting both generalist and specialist pollinators, as has been shown with plant invasions (Valdovinos et al. 2018; Parra‐Tabla and Arceo‐Gómez 2021).

To test whether the effects of varying nutrient types on forb–pollinator mutualisms differ across two main axes of pollination niche—forb family and flower color—we replicated a multifactorial fertilization experiment that crossed treatments of nitrogen, phosphorus, and potassium with micronutrients across three sites spanning a 150 km longitudinal transect in central California. The California Floristic Province is a global plant and pollinator biodiversity hotspot that can experience declines in local floral diversity and abundance under nutrient enrichment (Frankie et al. 2009; Harrison 2013; Eskelinen and Harrison 2015). We hypothesized that: (1) nitrogen addition will increase Asteraceae floral abundance, but decrease that of Fabaceae and other forb families, increasing visitation by late‐season bees that forage on Asteraceae. (2) Nitrogen addition will decrease the abundances of blue, pink, and green flowers, but increase that of purple and yellow flowers, due to shifts in dominance among flowering plant species decreasing visitation by flies. (3) Consequently, purple and yellow Asteraceae species that benefit from nitrogen addition will strongly contribute to plant–pollinator network structure by acting as centralized hubs that attract both generalist and specialist pollinators. Conversely, we predict that phosphorus or potassium with micronutrient addition in the absence of nitrogen will produce the opposite effect.

## 2 Materials and Methods

### 2.1 Study Sites

This research took place at three grassland sites in northern California that span a longitudinal rainfall gradient: the Sierra Foothill Research and Extension Center (39.24 N, −121.28 W, Mean Annual Precipitation: 936 mm, Mean Annual Temperature: 16.31°C), the University of California McLaughlin Reserve (38.86 N, −122.41 W, Mean Annual Precipitation: 936 mm; Mean Annual Temperature: 13.97°C), and the Hopland Research & Extension Center (39.01 N, −123.06 W, Mean Annual Precipitation: 1065 mm; Mean Annual Temperature: 13.24°C). We obtained permission for research activities at each site. All sites consisted of annual‐dominated grasslands in a matrix of oak savanna with a Mediterranean climate of cool wet winters and hot, dry summers. The sites were similar in their plant and pollinator species pools (Tables S1 and S2).

### 2.2 Nutrient Network Experiments

All three sites contained replicates of a randomized block, factorial experiment that used identical methods to manipulate fertilization for the global Nutrient Network (Borer et al. 2014; Seabloom et al. 2015). Each site had at least three randomized blocks of 5 m × 5 m plots with the following treatments. The experiment was composed of a factorial combination of three nutrient addition treatments: nitrogen (N), phosphorus (P), and potassium with micronutrients (Kμ) addition, creating eight treatment combinations: Control, N, P, Kμ, NPKμ, NKμ, PKμ, NP. Hopland and McLaughlin had three experimental blocks with these treatments, while Sierra Foothills had 5 experimental blocks. Application of experimental treatments began in 2008 and was assigned randomly to 5 × 5 m plots, the experimental unit.

The following nutrients were added annually to the fertilized plots: 10 g $N\;m^{-2}y^{-1}$ as slow release urea $(\left({\left({\mathrm{NH}}_{2}\right)}_{2}\mathrm{CO}\right))$, 10 g $P\;m^{-2}y^{-1}$ as triple‐super phosphate $\left(\mathrm{Ca}{\left(H_{2}PO_{4}\right)}_{2}\right)$, 10 g $K\;m^{-2}y^{-1}$ as potassium sulfate $\left(K_{2}SO_{4}\right)$. The plots receiving the potassium treatment received a one‐time addition of other micronutrients and macronutrients in the first year to include other nutrients essential to plant growth: 100 g m^−2^yr ^−1^ of a mixture of 15% iron (Fe), 14% sulfur (S), 1.5% magnesium (Mg), 2.5% manganese (Mn), 1% copper (Cu), 1% zinc (Zn), 0.2% boron (B), and 0.05% molybdenum (Mo). The control plots had no nutrients added.

### 2.3 Plant–Pollinator Observations

We surveyed each plot for pollinators (20‐min observations/plot) and forb abundance (# floral units/forb species/plot) at least three times over the course of the growing season (April–July) for 2 years, a minimum of 120 observer hours per plot per year in 2023 and 2024. Surveys occurred early, mid, and late season to capture a representative sample of species across the full flowering season from April to July in 2023 and 2024 (see Supporting Information Methods for list of sampling dates). The scale of our experimental design captures changes in the behavioral visitation of pollinators within a given site rather than population‐level changes across the landscape.

We surveyed each plot for floral resources and floral visitation by animals (hereafter referred to as ‘pollinator visitation’) (Veen et al. 2024). A floral visit occurred if an animal contacted the flower's reproductive structures. We recorded the morphospecies identity of the floral visitor and the floral species visited. Pollinator visitation surveys took place only on clear, calm, sunny, or partly sunny days and if flowering forbs were present in the plot. We collected floral abundance data for each plot within 10 days of the date a given pollinator survey occurred, so that floral abundance estimates would be temporally coupled to pollinator visitation data. We estimated floral abundance by counting the total number of floral units per flowering plant species in a given plot. A floral unit was defined as a 1 cm × 1 cm part of the flower (Veen et al. 2024).

Voucher specimens of each insect morphospecies were collected, pinned, and identified to species with the help of the Bohart Museum of Entomology (Davis, California, USA). Then, we classified pollinators into distinct morphospecies (Table S2). When collecting specimens during our surveys, we paused the timer and resumed timing once collection was complete. We collected at least 1–2 representative individuals of each nonsensitive taxa, collecting additional individuals for difficult to identify taxa. Field identifications were made by the lead author. We photographed sensitive taxa (*Bombus* spp. and Papilionidae spp.) instead of collecting vouchers. These taxa can be readily identified to species using photographs in the study region, and voucher photographs were made publicly available on iNaturalist. When multiple floral visitors were present at the same time, we were able to simultaneously record visitation with the help of digital photography. We ceased recording visits once a given individual pollinator left a plot. We aimed to collect individual visitors once they left a plot to minimize the impact of collection on visitation data.

### 2.4 Data Analysis

All data analyses were performed in R (v. 4.4.1) using the “glmmTMb” (Brooks et al. 2019), “sjPlot”(Lüdecke and Lüdecke 2015), and “Rmisc” (Hope et al. 2013) packages. Models were checked for normality of residuals and overdispersion when appropriate. We used mixed‐ effects models in order to draw statistical inferences from our experiment in relation to our hypotheses (Tredennick et al. 2021). For all analyses, we did pairwise post‐hoc analysis between treatments, families, and flower colors using Tukey HSD post‐hoc tests with the “eemeans” package (Lenth et al. 2024). Forb and pollinator abundance data per plot were summed across seasons within a given year by flower color and family. We performed rarefaction analyses for pollinator morphospecies using the “rarecurve” function in “vegan” (Figure S1) (Dixon 2003). Negative binomial models described below used a *z*‐distribution with infinite degrees of freedom.

To test our first hypothesis regarding nutrient effects on common floral families, we tested for interacting effects of treatment and family on floral abundance and pollinator visitation variables. We fit negative binomial generalized mixed‐effects models with interacting fixed effects of nutrient treatment and plant taxonomic family along with a nested random effect of site and experimental block on the response variables of floral abundance and pollinator visitation (# pollinator visits) totaled across all seasons within a given year (e.g., Floral_Abundance ~ Treatment + Family + Treatment × Family + (1|Site/block)). Both years of data were included in the same model. Introducing year as a random effect did not alter the outcome of the model and had near‐zero variance, so we did not include year in our final models. We ran Tukey HSD post hoc tests to examine whether response variables differed by each pairwise combination of nutrient treatment, each pairwise combination of floral family, within a given floral family, each pairwise combination of nutrient treatment, and within a given nutrient treatment between each pairwise combination of floral family. For a subset of common floral families whose abundance responded significantly to nutrient treatments—Fabaceae (legumes), Asteraceae (asters), Euphorbiaceae (spurges), Iridaceae (irises), and Geraniaceae (geraniums)—we further fit negative binomial mixed‐effects models that tested for fixed effects of treatment and family and a random effect of block nested within site on visitation for the following pollinator functional groups: flies (Diptera), beetles (Coleoptera), short‐tongued bees, and long‐tongued bees (e.g., #visits ~ Treatment + Family + (1|Site/block)). We classified bees (Anthophila) as short versus long‐tongued bees based on information obtained from the literature (LeBuhn 2013). We used Tukey HSD post hoc tests to examine how response variables differed by each pairwise combination of nutrient treatment and each pairwise combination of floral family. To further examine the effects of treatment and floral family on pollinator composition, we used redundancy analysis (RDA), Permanovas with Bray–Curtis dissimilarity index and mvabund multivariate abundance analyses to test for effects of treatment and forb family on pollinator morphospecies composition and pollinator functional group composition using the “vegan” and “mvabund” packages (Dixon 2003; Wang et al. 2012). We further used mvabund analyses to test for differences in pollinator morphospecies multivariate abundance by each pairwise combination of our five focal families. We used an NMDS visualization to visualize the results of the Permanovas.

To test our second hypothesis regarding nutrient effects by flower color, we used a color wheel to classify flower colors into broad categories of pink, purple, yellow, blue, white, orange, red, and green (Kearns and Inouye 1993; Szigeti et al. 2020). Because pollinator color vision can be quite complex and variable across insect taxa but has overlap with how vertebrates perceive color (Chittka and Menzel 1992; Rausher 2008; Song and Lee 2018; Paine et al. 2019; Garcia et al. 2020; Dyer et al. 2021) and our question of interest applies to the whole floral visitor community, we chose this more classic approach of using a color wheel as a proxy for pollinator perceptions of color differences (Kearns and Inouye 1993). With the exceptions of a few model insect taxa, detailed color perception information is lacking for most insect taxa, with much information obtained from highly controlled laboratory experiments rather than from real‐world field settings (Song and Lee 2018; Garcia et al. 2020). Thus, we used human color perception as an integrator across differing insect perceptions of color (Szigeti et al. 2020). All color classifications were made by the lead author's perceptions of a color wheel to standardize color classifications across perceptions by a single human observer (Kearns and Inouye 1993; Szigeti et al. 2020).

We fit negative binomial generalized mixed‐effects models with interacting fixed effects of nutrient treatment and flower color and nested random effect of site and experimental block for the response variables of floral abundance and pollinator visitation (# pollinator visits) totaled across all seasons within a given year (e.g., Floral_Abundance ~ Treatment + Color + Treatment × Color + (1|Site/block)). Both years of data were included in the same model. We ran Tukey HSD post hoc tests to examine whether response variables differed by each pairwise combination of nutrient treatment, each pairwise combination of flower color, within a given flower color, each pairwise combination of nutrient treatment, and within a given nutrient treatment between each pairwise combination of flower color. For a subset of flower colors whose abundances responded significantly to nutrient treatments—blue, yellow, purple, pink, and green—we further fit negative binomial mixed‐effects models that tested for fixed effects of treatment and family and a random effect of block nested within site on visitation for the following pollinator functional groups: flies (Diptera), beetles (Coleoptera), short‐tongued bees, and long‐tongued bees (e.g., #visits ~ Treatment + Color + (1|Site/block)). We used Tukey HSD post hoc tests to examine how response variables differed by each pairwise combination of nutrient treatment and each pairwise combination of flower color. To further examine the effects of treatment and flower color on pollinator composition, we used redundancy analysis (RDA), Permanovas with Bray–Curtis dissimilarity, and mvabund multivariate abundance analyses to test for effects of treatment and flower color on pollinator morphospecies composition and pollinator functional group composition using the “vegan” and “mvabund” packages (Dixon 2003; Wang et al. 2012). We used an NMDS visualization to graph the results of the Permanovas.

To test for whether plant provenance (native to central Califiornia vs. exotic) further modulated changes in floral abundance under nutrient enrichment, we added a covariate of plant provenance to our negative binomial model (e.g., Floral_Abundance ~ Treatment*Color*Provenance + (1|Site/block)). We used Tukey HSD post hoc tests to examine how differences in floral abundance between nutrient treatments varied by each combination of flower color and provenance.

To test our third hypothesis regarding network structure, we constructed plant–pollinator networks for each site that pooled together data from all years and treatments. We used the “bipartite” package in R to calculate the individual contribution to network nestedness for each plant species (Dormann et al. 2009). Individual contribution to network nestedness measures how strongly a plant species contributes to the overall structure of the network by attracting both generalist and specialist pollinators (Dormann et al. 2009). A network is considered highly nested if both specialist and generalist pollinators visit generalist plants; nestedness is associated with the stability of mutualistic networks (Bascompte et al. 2003; Bastolla et al. 2009). We used the following formula to compute two‐tailed *p*‐values for individual contribution to nestedness *z*‐scores: *p*‐value = 2*(1 − Φ(∣*z*∣)). Φ is the cumulative distribution function of a standard normal distribution. A *p* value greater than the alpha level of 0.05 indicates that a plant species contributes more strongly to the nested structure of the network than the random null model used by the contribution to nestedness function that controls for the effect of differences in network degree (number of interactions a given plant node has).

We used the “igraph” package to calculate the betweenness centrality of each plant species in the networks (Csardi et al. 2025). Betweenness centrality measures the relative number of shortest paths between species nodes for a given species (Csardi et al. 2025), a measure of how much of a hub for pollinators a given plant species is within the network. A plant species with high betweenness centrality would act as a centralized, important hub in the network that interacts with many other species.

To further test the relationship between individual nestedness contribution and floral abundance, measured independently of the network, we fit a linear mixed‐effects model to test for fixed effects of total floral abundance, floral family, and flower color, with a random effect of site on individual contribution to nestedness by plant species. We then fit additional linear mixed‐effects models that tested for a fixed effect of total floral abundance and a random effect of site on the nestedness contribution of each individual family and flower color. We summed floral abundance across all plots and years for a given plant species, family, or floral color within a given site to match the level of aggregation of network data.

We surveyed floral abundance for 88 forb species in total across our three sites, 43 species of which we observed being visited by pollinators (Table S1). We observed a total of 73 pollinator morphospecies across our three sites, and 1098 individual floral visitors to forbs (Table S2). At McLaughlin, we observed a total of 38 pollinator morphospecies visiting 14 plant species. At Hopland, we observed a total of 37 pollinator morphospecies visiting 16 unique plant species. At Sierra Foothills, we observed a total of 46 pollinator morphospecies visiting 28 unique plant species. Common floral visitors included halictid bees, apid bees, megachilid bees, bombyliid flies, syrphid flies, and *Listrus* beetle species (Table S2).

## 3 Results

### 3.1 Flower Families

#### 3.1.1 Effect of Nutrients on Focal Plant Families

**FIGURE 1:**
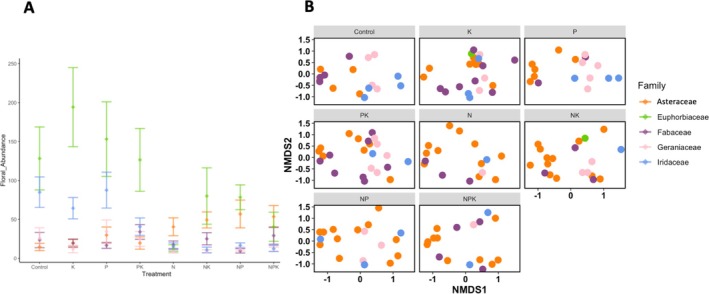
The effects of nutrient treatment on (A) floral abundance and (B) pollinator morphospecies composition for Asteraceae (aster family, shaded orange), Euphorbiaceae (spurge family, shaded green), Fabaceae (legume family, shaded purple), Geraniaceae (geranium family, shaded pink), and Iridaceae (iris family, shaded blue). Intervals show mean and standard error. Control refers to unfertilized control treatment, K refers to the addition of potassium with micronutrients, P refers to phosphorus addition, and N refers to nitrogen addition. Bars show mean with standard error. Statistical results that correspond to Figure 1A are provided in Table S3.

In support of our first hypothesis, nitrogen addition increased Asteraceae floral abundance but decreased the floral abundances of Fabaceae, Euphorbiaceae, Iridaceae, and Geraniaceae (Figures 1A and S2, Table S3). Adding nitrogen (*z* = −4.734, *p* = 0.0001), nitrogen combined with potassium with micronutrients (*z* = −5.602, *p* < 0.0001), nitrogen combined with phosphorus (*z* = −4.963, *p* < 0.0001), and combined nitrogen, phosphorus, and potassium with micronutrients (*z* = −5.107, *p* < 0.0001) all increased Asteraceae floral abundance relative to the unfertilized control. In contrast, adding nitrogen decreased the floral abundance of Euphorbiaceae (*z* = 3.532, *p* = 0.0098), Iridaceae (*z* = 3.836, *p* = 0.0031), and Geraniaceae (*z* = 3.034, *p* = 0.0496) relative to the control. Likewise, the addition of nitrogen and phosphorus in combination decreased the floral abundance of Fabaceae relative to the control (*z* = 3.632, *p* = 0.0068).

Consequently, nitrogen increased the abundance of Asteraceae relative to Fabaceae, Euphorbiaceae, Iridaceae, and Geraniaceae. While Asteraceae and Fabaceae floral abundance did not differ in the control treatment, Asteraceae had higher floral abundance than Fabaceae under nitrogen addition (*z* = 3.730, *p* = 0.0266), combined nitrogen and potassium with micronutrients addition (*z* = 3.815, *p* = 0.0162), and combined nitrogen and phosphorus addition (*z* = 6.046, *p* < 0.0001). Likewise, while Asteraceae and Geraniaceae floral abundance did not differ in the control (*z* = −2.010, *p* = 0.8831), Asteraceae had higher floral abundance than Geraniaceae under nitrogen addition (*z* = 5.568, *p* < 0.0001), combined nitrogen and potassium with micronutrients addition (*z* = 6.707, *p* < 0.0001), combined nitrogen and phosphorus addition (*z* = 5.362, *p* < 0.0001), and combined nitrogen, phosphorus, and potassium with micronutrients addition (*z* = 4.181, *p* = 0.0034).

Moreover, Iridaceae had higher floral abundance than Asteraceae in the control (*z* = −4.720, *p* = 0.0004), while Asteraceae had higher floral abundance than Iridaceae under combined nitrogen and potassium with micronutrient addition (*z* = 3.798, *p* = 0.0172). Similarly, Euphorbiaceae had higher floral abundance than Asteraceae in the control treatment (*z* = −4.488, *p* = 0.0011) and under the addition of potassium with micronutrients (*z* = −4.413, *p* = 0.0017), but Euphorbiaceae and Asteraceae did not differ in abundance under nitrogen addition (*z* = 2.666, *p* = 0.4538).

### 3.2 Differences in Pollinators Among Floral Families

Nutrient treatment explained pollinator composition (RDA df = 7, F = 5.7431, *p* = 0.001). Nutrient treatment did not directly affect pollinator visitation (total number of visits per plot summed for all flowers within a given family across all surveys within a given year) except by increasing total pollinator visitation to Ranunculaceae under combined nitrogen and potassium with micronutrient addition than with the addition of potassium and micronutrients alone (*z* = −3.27, *p* = 0.0243). Pollinator multivariate abundance (mvabund glms, df = 14, deviance = 1054, *p* = 0.001) and multivariate functional group abundance (mvabund glms, df = 13, deviance = 347.4, *p* = 0.001) significantly differed by floral family. Likewise, pollinator community composition as measured by the Bray–Curtis Similarity Index significantly varied by floral family (PERMANOVA, df = 14, F = 3.44, *p* = 0.001). In support of our first hypothesis, Asteraceae, which increased in floral abundance under nitrogen addition, differed in pollinator composition from Fabaceae, Geraniaceae, Euphorbiaceae, and Iridaceae, all of which decreased in floral abundance under nitrogen enrichment (Figure 1B).

Asteraceae and Fabaceae did not differ in total number of pollinator visits (*z* = −0.031, *p* = 1.0000, Figure S3), long‐tongued bee visits (*z* = −1.749, *p* = 0.4037), short‐tongued bee visits (*z* = 1.387, *p* = 0.6359), beetle visits (*z* = 2.546, *p* = 0.0807), and fly visits (*z* = 0.508, *p* = 0.9866), measured by the total number of visits for a given pollinator functional group per plot summed for all flowers within a given family across all surveys within a given year. However, Fabaceae and Asteraceae significantly differed in multivariate pollinator abundance (mvabund glms, df = 1, deviance = 132.4, *p* = 0.001). At the morphospecies level, Asteraceae but not Fabaceae was visited by the late season leaf cutter bee *Megachile apicalis* (mvabund glms, df = 1, deviance = 12.814, *p* = 0.008).

Asteraceae had higher total pollinator visits (*z* = 6.110, *p* < 0.0001), higher long‐tongued bee visits (*z* = 5.108, *p* < 0.0001), higher short‐tongued bee visits (*z* = 3.701, *p* = 0.0020), and higher beetle visits (*z* = 3.036, *p* = 0.0203) than Geraniaceae. Asteraceae and Geraniaceae did not differ in fly visitation (*z* = −2.370, *p* = 0.1236). Asteraceae and Geraniaceae differed in pollinator multivariate abundance (mvabund glms, df = 1, deviance = 150.9, *p* = 0.001). Asteraceae but not Geraniaceae was visited by the leaf cutter bee *Megachile apicalis* (mvabund glms, df = 1, deviance = 9.645, *p* = 0.034), the western honeybee *Apis mellifera* (mvabund glms, df = 1, deviance = 26.196, *p* = 0.001), and the yellow‐faced bumblebee *Bombus vosnesenskii* (mvabund glms, df = 1, deviance = 11.728, *p* = 0.015).

Asteraceae did not differ in total pollinator visits from Euphorbiaceae (*z* = 3.048, *p* = 0.1494). Euphorbiaceae, however, received a higher number of fly visits than Asteraceae (*z* = 3.247, *p* = 0.0103). Asteraceae and Euphorbiaceae did not differ in long‐tongued bee visits (*z* = 0.004, *p* = 1.0000), short‐tongued bee visits (*z* = 0.004, *p* = 1.0000), and beetle visits (*z* = 0.002, *p* = 1.0000). Euphorbiaceae and Asteraceae significantly differed in multivariate pollinator abundance (mvabund glms, df = 1, deviance = 77.4, *p* = 0.004). An unidentified syrphid fly morphospecies (Syrphidae) visited Euphorbiaceae more than Asteraceae (mvabund glms, df = 1, deviance = 33.77, *p* = 0.001).

Asteraceae and Iridaceae did not differ in total pollinator visits (*z* = 1.667, *p* = 0.9554), long‐tongued bee visits (*z* = 1.081, *p* = 0.8166), short‐tongued bee visits (*z* = −0.004, *p* = 1.000), or beetle visits (*z* = 1.911, *p* = 0.3112). However, Iridaceae had more fly visits than Asteraceae (*z* = 3.073, *p* = 0.0181). Iridaceae and Asteraceae differed in pollinator multivariate abundance (mvabund glms, df = 1, deviance = 144.7, *p* = 0.001). Asteraceae received more *Apis mellifera* visits than Iridaceae (mvabund glms, df = 1, deviance = 17.484, *p* = 0.003). The white‐browed smoothwing fly *Scaeva affinis* visited Iridaceae but not Asteraceae (mvabund glms, df = 1, deviance = 13.691, *p* = 0.007) as did the bee *Anthophora californica* (mvabund glms, df = 1, deviance = 10.096, *p* = 0.035), while the yellow‐faced bumblebee *Bombus vosnesenskii* visited Asteraceae but not Iridaceae (mvabund glms, df = 1, deviance = 9.654, *p* = 0.049).

### 3.3 Flower Color

#### 3.3.1 Effect of Nutrients on Plants by Flower Color

**FIGURE 2:**
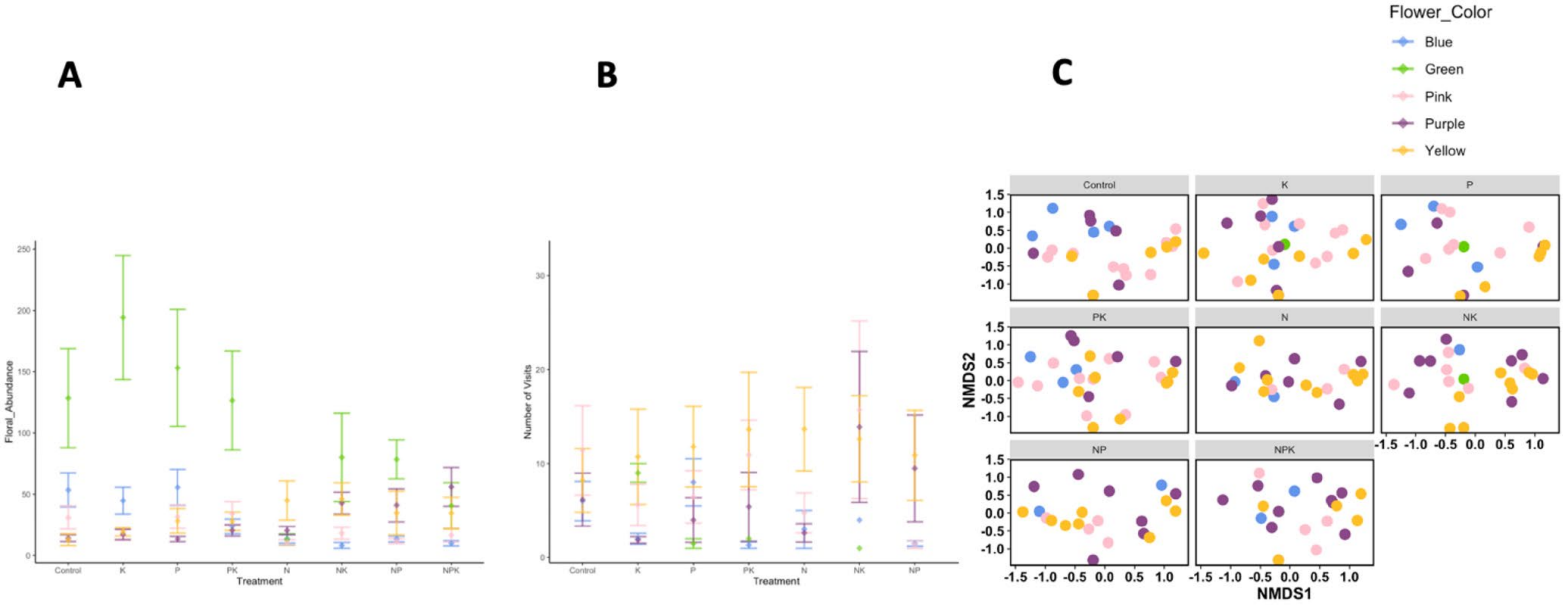
The effects of nutrient treatment on (A) floral abundance, (B) total pollinator visitation and (C) pollinator morphospecies composition for blue flowers (shaded blue), yellow flowers (shaded yellow), green flowers (shaded green), pink flowers (shaded pink), and purple flowers (shaded purple). Intervals show mean and standard error. Control refers to unfertilized control treatment, K refers to the addition of potassium with micronutrients, P refers to phosphorus addition, and N refers to nitrogen addition. Bars show mean with standard error.

Consistent with our second hypothesis, nitrogen addition decreased the floral abundance of blue, green, and pink flowers, while increasing the abundance of yellow and purple flowers (Figures 2A and S4, Table S4). Adding nitrogen decreased flower abundance relative to the unfertilized control of blue flowers (*z* = 3.607, *p* = 0.0075), pink flowers (*z* = 4.272, *p* = 0.0005), and green flowers (*z* = 3.343, *p* = 0.0188), but increased the abundance of yellow flowers (*z* = 4.572, *p* = 0.0001). Adding nitrogen in combination with potassium with micronutrients (*z* = 3.357, *p* = 0.0180), in combination with phosphorus (*z* = −3.195, *p* = 0.0304), and in combination with both potassium with micronutrients and phosphorus (*z* = 4.188, *p* = 0.0007) increased the abundance of purple flowers. Nitrogen addition did not affect the abundance of white flowers (*z* = 2.352, *p* = 0.2657), nor orange flowers (*z* = 1.504, *p* = 0.8057).

Consequently, nitrogen addition shifted the relative abundance of flower colors to increase the abundance of yellow and purple flowers relative to green, blue, and pink flowers. Adding nitrogen reduced the relative abundance of green flowers, causing them to be equally abundant as yellow and purple flowers. Green flowers were more abundant than purple flowers under ambient conditions (*z* = 4.117, *p* = 0.0008), the addition of potassium with micronutrients (*z* = 4.714, *p* = 0.0001), and under phosphorus addition (*z* = 4.823, *p* < 0.0001). However, nitrogen addition equalized the abundance of green and purple flowers (*z* = −1.017, *p* = 0.9721). Likewise, green flowers were more abundant than yellow flowers under the control (*z* = 4.472, *p* = 0.0002), the addition of potassium with micronutrients (*z* = 4.365, *p* = 0.0003), and phosphorus addition (*z* = 3.318, *p* = 0.0158) but not under nitrogen addition (*z* = 2.506, *p* = 0.1925).

Moreover, blue flowers were more abundant than purple flowers under the control (*z* = 3.852, *p* = 0.0023) and under phosphorus addition (*z* = 4.315, *p* = 0.0003) but not under nitrogen addition (*z* = −1.098, *p* = 0.9575). In fact, purple flowers became more abundant than blue flowers under the combined addition of nitrogen and potassium with micronutrients (*z* = 4.184, *p* = 0.0006), under combined nitrogen and phosphorus addition (*z* = 3.267, *p* = 0.0242), and under the combined addition of nitrogen, phosphorus and potassium with micronutrients (*z* = −4.040, *p* = 0.0010). Similarly, blue flowers were more abundant than yellow flowers under the control (*z* = 4.308, *p* = 0.0003), but yellow flowers became more abundant than blue flowers under nitrogen addition (*z* = 3.459, *p* = 0.0127), and combined nitrogen and potassium with micronutrients addition (*z* = 4.922, *p* < 0.0001).

Likewise, pink flowers did not differ in abundance from purple flowers under the control (*z* = 2.824, *p* = 0.0708) and were more abundant than purple flowers under phosphorus addition (*z* = 3.150, *p* = 0.0272), but purple flowers became more abundant than pink flowers when adding nitrogen in combination with potassium with micronutrients (*z* = 3.012, *p* = 0.0415), when adding nitrogen in combination with phosphorus (*z* = 4.124, *p* = 0.0010), and when adding combined nitrogen, phosphorus, and potassium with micronutrients (*z* = 4.025, *p* = 0.0011). Similarly, pink flowers were more abundant than yellow flowers under the control (*z* = 3.545. *p* = 0.0072), but yellow flowers became more abundant than pink flowers under the addition of nitrogen (*z* = 5.056, *p* < 0.0001) and nitrogen in combination with potassium with micronutrients (*z* = 4.132, *p* = 0.0007).

Provenance (native vs. exotic) further interacted with flower color to modulate how floral abundances responded to nutrient additions. All blue and green flower species were native plant species, while both native and exotic plant species had yellow, pink, and purple flowers. Thus, decreases in the floral abundances of green and blue flowers under nitrogen enrichment (green: *z* = 3.456, *p* = 0.0128, blue: *z* = 3.737, *p* = 0.0046) reflect decreases in entirely native wildflower floral abundances. Decreases in the floral abundances of pink flowers under nitrogen addition, however, reflect decreases in exotic pink flowers (*t* = 4.354, *p* = 0.0004), but not native pink flowers (*t* = 1.910, *p* = 0.5438). Both native yellow flowers and exotic yellow flowers increased in floral abundance under nitrogen addition compared to ambient conditions (exotic: *z* = 3.758, *p* = 0.0043; native *z* = −3.096, *p* = 0.0412). However, the increases in the abundance of purple flowers under the addition of nitrogen in combination with phosphorus and potassium with micronutrients only reflect increases in exotic purple flowers (exotic: *z* = 4.338, *p* = 0.0004; native: *z* = 0.788, *p* = 0.9938).

#### 3.3.2 Differences in Pollinators Among Flower Colors

In support of our second hypothesis, flower color mediated pollinator responses to nutrient addition (Figures 2B and S5). Nitrogen additions increased total pollinator visitation to purple flowers, while decreasing total pollinator visitation to pink flowers.

Pollinator visitation to pink flowers decreased under combined nitrogen and phosphorus addition relative to the control (*z* = −3.373, *p* = 0.0170) and to combined phosphorus and potassium addition (*z* = −3.395, *p* = 0.0158). In contrast, combined nitrogen and phosphorus addition increased pollinator visitation to purple flowers relative to potassium addition (*z* = 3.111, *p* = 0.0394), as did combined nitrogen and potassium with micronutrients addition (*z* = 3.926, *p* = 0.0022) and combined nitrogen, phosphorus, and potassium with micronutrient addition (*z* = 3.331, *p* = 0.0196). Consequently, under combined nitrogen and phosphorus addition, pollinators visited more purple flowers than pink flowers (*z* = 3.012, *p* = 0.0415).

Pollinator responses to eutrophication, flower color, and their interactions further varied by pollinator functional group, as measured by the total number of visits for a given pollinator functional group per plot summed for all flowers within a given color across all surveys within a given year. Within purple flowers, the treatment that combined the addition of potassium with micronutrients, nitrogen, and phosphorus received more long‐tongued bee visits than the treatment where only potassium with micronutrients was added (*z* = 3.060, *p* = 0.0459). Within the potassium with micronutrients treatment, long‐tongued bees visited pink flowers more than purple flowers (*z* = 2.773, *p* = 0.0441).

Other pollinator preferences did not vary with nutrients. Short‐tongued bees and beetles visited more yellow than pink flowers (short‐tongued bees: *z* = −2.983, *p* = 0.0239; beetles: *z* = −2.783, *p* = 0.0429). Flies visited green flowers more than purple flowers (*z* = 4.166, *p* = 0.0003) and blue flowers more than purple flowers (*z* = 3.597, *p* = 0.0030).

Pollinator multivariate abundance significantly differed by flower color for all pairwise combinations of colors (mvabund glms, df = 6, deviance = 774.1, *p* = 0.001) as did pollinator community composition as measured by Bray–Curtis Dissimilarity (PERMANOVA, df = 6, F = 4.372, *p* = 0.001) (Figure 2C). Short‐tongued leaf‐cutter bees *Megachile apicalis* visited only yellow flowers (and an individual white flower) (mvabund glms, df = 1, deviance = 52.098, *p* = 0.001). The orange sulfur butterfly *Colias eurytheme* visited only yellow flowers (mvabund glms, df = 1, deviance = 24.787, *p* = 0.013). Pollinator abundance significantly differed by flower color for several bee species: western honeybee *Apis mellifera* visited mostly yellow flowers (mvabund glms, df = 1, deviance = 45.095, *p* = 0.002), yellow‐faced bumblebee *Bombus vosnesenskii* visited mostly pink and purple and some yellow flowers (mvabund glms, df = 1, deviance = 25.717, *p* = 0.013), black‐tailed bumblebee *Bombus melanopygus* visited mostly pink flowers (mvabund glms, df = 1, deviance = 19.567, *p* = 0.039), and the blue mason bee *Osmia cara* visited mostly pink and purple flowers (mvabund glms, df = 1, deviance = 28.984, *p* = 0.013). Among individual fly morphospecies, the white‐browed smoothwing *Scaeva affinis* , which visited only blue (and an individual pink flower) (mvabund glms, df = 1, deviance = 19.203, *p* = 0.042), the common beefly *Bombylius major* visited mostly orange flowers (mvabund glms, df = 1, deviance = 23.072, *p* = 0.013), and an unidentified syrphid fly morphospecies (Syrphidae) visited mostly green flowers (mvabund glms, df = 1, deviance = 42.633, *p* = 0.002).

### 3.4 Network Contributions

**FIGURE 3:**
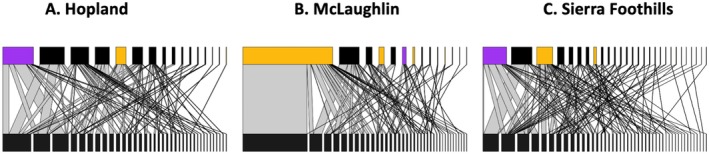
Plant–pollinator networks for three sites (A) Hopland, (B) McLaughlin, and (C) Sierra Foothills listed from left to right in order of increasing aridity. Each box in the top row represents a unique plant species observed in the network, while each box in the bottom row represents a unique pollinator species observed in the network, with interactions representing the lines between them. The thickness of the boxes is in proportion to the frequency with which that group or interaction was observed in the network. Yellow Asteraceae are shaded in yellow, and purple Asteraceae are shaded in purple.

In support of our third hypothesis, yellow and purple asters contributed strongly to network structure as centralized hubs for pollinators by attracting both generalist and specialist pollinators (Figure 3, Tables 1, S5 and S6). In particular, the native yellow aster *Agoseris heterophylla* , the exotic yellow aster *Centaurea solstitialis* , and the exotic purple aster *Carduus pycnocephalus* had high individual contributions to network nestedness (ability to attract a diversity of both specialist and generalist pollinators) and betweenness centrality (ability to act as a bridge that shortens the connections between different plant and pollinator species nodes in the network) (Table 1).

**TABLE 1:** Core plant hubs in the plant–pollinator network.

Site	Network metric	Plant species	Family	Color	Provenance	Metric value
Sierra foothills	Individual nestedness contribution	***Agoseris heterophylla***	Asteraceae	Yellow	Native	*z* = 4.40; *p* = 1.083 × 10^−5^
		***Carduus pycnocephalus***	Asteraceae	Purple	Exotic	*z* = 3.01; *p* = 0.0026
		*Amsinckia menziesii*	Boraginaceae	Orange	Native	*z* = 2.03; *p* = 0.042
	Betweenness centrality	***Agoseris heterophylla***	Asteraceae	Yellow	Native	986.63
		***Carduus pycnocephalus***	Asteraceae	Purple	Exotic	572.08
		*Hypericum perforatum*	Hypericaceae	Yellow	Exotic	512.47
McLaughlin	Individual nestedness contribution	***Centaurea solstitialis***	Asteraceae	Yellow	Exotic	*z* = 3.45; *p* = 0.00056
		*Delphinium hesperium*	Ranunculaceae	Pink	Native	*z* = 2.28; *p* = 0.023
		*Geranium dissectum*	Geraniaceae	Pink	Exotic	*z* = 1.65; *p* = 0.0989
	Betweenness centrality	***Centaurea solstitialis***	Asteraceae	Yellow	Exotic	657.19
		*Geranium dissectum*	Geraniaceae	Pink	Exotic	344.91
		*Delphinium hesperium*	Ranunculaceae	Pink	Native	262.53
Hopland	Individual nestedness contribution	*Sisyrinchium bellum*	Iridaceae	Blue	Native	*z* = 4.15; *p* = 3.33 × 10^−5^
		*Dichelostemma congestum*	Asparagaceae	Blue	Native	*z* = 2.11; *p* = 0.035
		***Carduus pycnocephalus***	Asteraceae	Purple	Exotic	*z* = 1.91; *p* = 0.056
	Betweenness centrality	*Sisyrinchium bellum*	Iridaceae	Blue	Native	643.43
		*Dichelostemma congestum*	Asparagaceae	Blue	Native	325.32
		***Carduus pycnocephalus***	Asteraceae	Purple	Exotic	280.92

*Note:* For networks encapsulating plant–pollinator interaction data pooled across all treatments, plots, and sampling dates for each site, the three plant species listed contribute the most strongly to individual plant species contribution to network nestedness and betweenness centrality. Individual contribution to network nestedness measures how strongly a plant species contributes to the network's structure by attracting both generalist and specialist pollinators. Betweenness centrality measures how much a plant species node lies on the shortest path between other network nodes, acting a bridge that connects different species within the network. Taken together, both metrics indicate how strongly a given plant species acts a core hub for pollinators within the network. Additional information on each plant species' taxonomic family, flower color, and provenance (native vs. exotic to California) are provided. Plant species that are purple and yellow in color and in Asteraceae are bolded.

Individual plant contribution to network nestedness positively correlated with floral abundance at the level of individual plant species (df = 1, *z* = 5.529, *p* = 3.23 × 10^−8^), family (df = 1, *z* = 3.509, *p* = 0.000451), and flower color (df = 1, *z* = 2.117, *p* = 0.0342). Plant species in Euphoribacae contributed less strongly to plant species nestedness contributions than plant species in Compositae (SE = 0.720, df = 120, *t* = −5.258, *p* = 0.0001), Fabaceae (SE = 0.744, df = 120, *t* = −4.144, *p* = 0.0054), Geranianceae (SE = 0.750, df = 120, *t* = −4.013, *p* = 0.0086), and Iridaceae (SE = 0.782, df = 120, *t* = −5.284, *p* = 0.0001). Likewise, plant species with green flowers contributed less strongly to network nestedness than blue flowers (SE = 0.771, df = 128, *t* = −5.109, *p* < 0.0001), pink flowers (SE = 0.732, df = 128, *t* = −4.852, *p* = 0.0001), purple flowers (SE = 0.741, df = 128, *t* = −5.586, *p* < 0.0001), and yellow flowers (SE = 0.736, df = 128, *t* = −5.634, *p* < 0.0001).

## 4 Discussion

**FIGURE 4:**
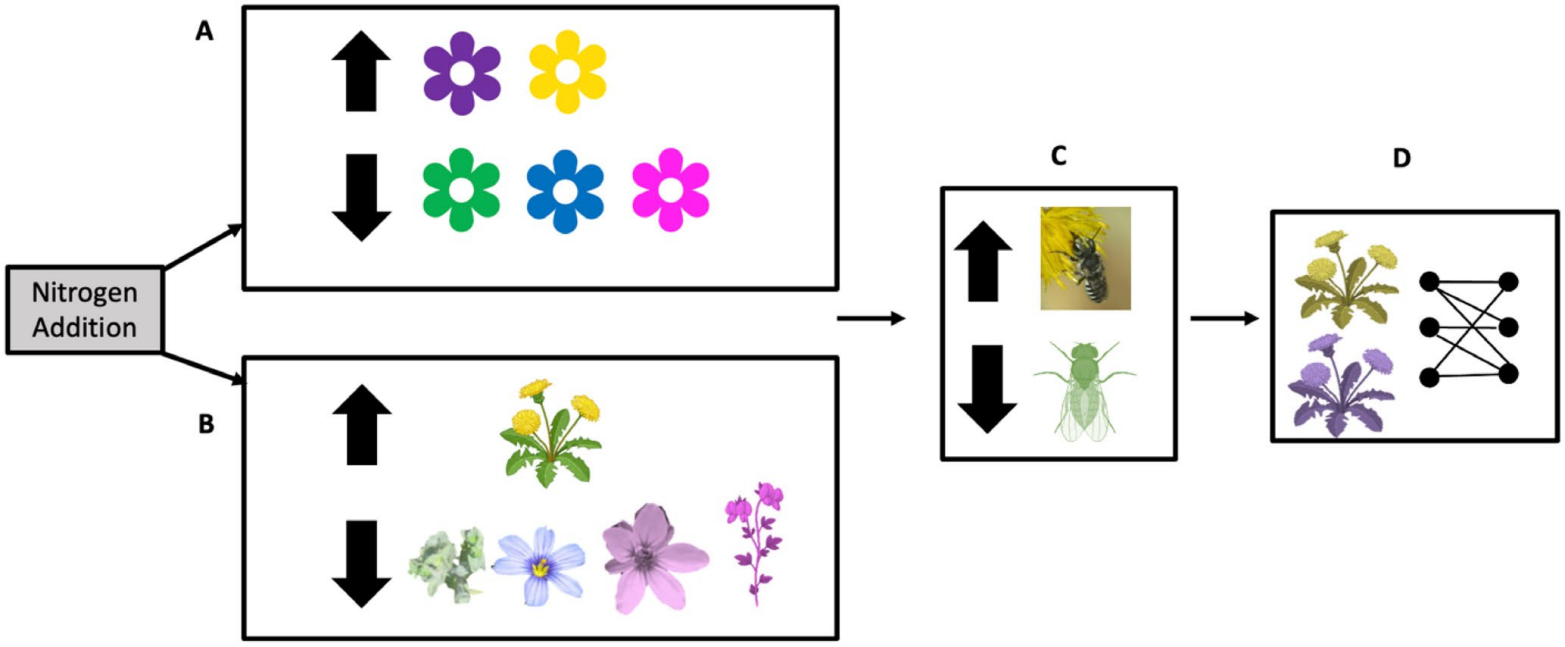
Summary of key findings. Nitrogen addition (A) increased the abundances of purple and yellow flowers but decreased the abundances of green, blue, and pink flowers; and (B) increased the abundance of Asteraceae but decreased the abundances of Euphorbiaceae, Iridaceae, Geraniaceae, and Fabaceae. Changes in the relative abundances of floral color and family mediated compositional shifts in the pollinator community as (C) increases in yellow Asteraceae increased visitation by the exotic, late season leaf cutter bee *Megachile apicalis* and decreases in blue Iridaceae and green Euphorbiaceae decreased fly visitation. (D) Purple and yellow Asteraceae species acted as central hubs in the plant–pollinator network. *Image icons of fly, legume, and aster*. BioRender. Retrieved from https://biorender.com.

Consistent with our first hypothesis, adding nitrogen increased the floral abundance of Asteraceae, while decreasing that of Fabaceae, Iridaceae, Geraniaceae, and Euphorbiaceae. Consistent with our second hypothesis, adding nitrogen increased the abundances of native and exotic yellow and exotic purple flowers, while decreasing the abundances of native blue, native green, and exotic pink flowers (Figure 4). Adding phosphorus or potassium with micronutrients in the absence of nitrogen, however, produced the opposite effects on family and color responses. Changes in flower family and color explained changes in pollinator composition. Western honeybee ( *Apis mellifera* ) and leafcutter bee ( *Megachile apicalis* ) visitation to yellow Asteraceae increased, while fly visitation to blue Iridaceae and green Euphorbiaceae decreased. Consistent with our third hypothesis, purple and yellow Asteraceae, which increased under nitrogen addition, played a core role in the plant–pollinator network as centralized hubs that supported both specialist and generalist pollinators.

Two abundant, functionally important grassland forb families—Asteraceae and Fabaceae—had contrasting responses to nutrient enrichment. These contrasting responses may arise because adding nitrogen allows other plant species that are limited by nitrogen, such as species in Asteraceae, to outcompete nitrogen‐fixing Fabaceae (Tognetti et al. 2021). Phosphorus and potassium with micronutrient addition in the absence of nitrogen increased Fabaceae and decreased Asteraceae floral abundance, while adding nitrogen had the opposite effect. This finding is consistent with prior work showing that phosphorus addition can decrease Asteraceae abundance (Tilman et al. 1999) and increase Fabaceae abundances (Tognetti et al. 2021; Nelson et al. 2025). At the global scale, however, nitrogen addition decreased Asteraceae diversity and abundance (Nelson et al. 2025). This difference in nitrogen response in Asteraceae between our California sites and the global Nutrient Network dataset may occur because many of the dominant Asteraceae species at our sites are invasive species (Seabloom et al. 2006, 2009). Although Asteraceae and Fabaceae supported similar functional groups of pollinators, they differed in their pollinator species composition, suggesting that differences in the type of nutrient between nitrogen and phosphorus or potassium could produce shifts in pollinator composition but may maintain overall ecosystem functions and services. This finding is consistent with prior work that found that shifts between nitrogen‐philous versus nitrogen‐phobic plants under nitrogen enrichment can drive shifts in pollinator species assemblages over longer time scales (Carvalheiro et al. 2020). Moreover, Asteraceae species in our study tended to flower primarily in mid to late season, while Fabaceae species tended to flower early to mid season, suggesting that these shifts toward Asteraceae at the expense of Fabaceae (and vice versa) could produce phenological gaps in floral resources. These gaps in phenology could pose a problem for supporting pollinators over the full growing season, particularly for species like bumblebees that have long flight periods (Ogilvie et al. 2017; Timberlake et al. 2019).

The abundance of flowers in Euphorbiaceae, Iridaceae, and Geraniaceae decreased with nitrogen addition but increased with phosphorus addition in the absence of nitrogen. Similarly to Fabaceae, these floral families may be less nitrogen‐limited and consequently get outcompeted by faster‐growing, weedy Asteraceae and grasses under nitrogen addition. Thus, the responses of Euphorbiaceae, Iridaceae, and Geraniaceae matched that of Fabaceae, but contrasted with that of Asteraceae. Euphorbiaceae and Iridaceae provided key floral resources for flies in our study, suggesting that decreases in Euphorbiaceae and Iridaceae under nitrogen enrichment may deplete floral resources for flies, while phosphorus addition may enhance them. In contrast, nitrogen addition in combination with phosphorus and potassium increased fly abundance and visitation in a European meadow (Veen et al. 2024). These contrasting results may reflect differences in fly specialization. In the European meadow, the flies were mostly generalist hoverflies that increased in visitation in response to increasing overall flower abundance under nutrient addition (Veen et al. 2024), while in the California grasslands, the fly species we observed showed preferences toward certain flower colors and families (i.e., green Euphorbiaceae, blue Iridaceae) that declined in abundance under nitrogen addition.

The addition of nitrogen overwhelmed any effects of phosphorus or potassium with micronutrients on forb families, consistent with prior findings (Fay et al. 2015; Tognetti et al. 2021), and our results demonstrate that this impact extends to flower colors and pollinator responses, as well. This is concerning, given that nitrogen pollution is common worldwide (Li et al. 2022). Not only can nutrient enrichment drive declines in the diversity and abundance of forbs and their pollinators (Valliere et al. 2020; Veen et al. 2024; Nelson et al. 2025) but it can also engender functional shifts in plant and pollinator community composition (Carvalheiro et al. 2020; Wilcots et al. 2021). Thus, the relative ratios of these different nutrients may drive forb community responses (Fay et al. 2015; Wilcots et al. 2021) and consequently that of pollinators.

Plant communities with a variety of different flower colors can support greater pollinator diversity (Shrestha et al. 2019; Phillips et al. 2020), and our results demonstrate that nutrient enrichment alters the relative abundance of flower colors. Consistent with prior findings (Dyer et al. 2021), blue flowers declined under nitrogen enrichment, while purple and yellow flowers increased, causing pollinators, particularly bees, to shift to visit purple flowers over blue flowers. Green and blue flowers, visited by flies (Dellinger 2020; del Valle et al. in press), declined under nitrogen enrichment, pointing to the likelihood of future diversity loss for flies.

Moreover, under nitrogen addition, decreases in the floral abundances of native blue flowers, native green flowers, and pink exotic flowers and increases in the floral abundances of native yellow flowers, exotic yellow flowers, and exotic purple flowers may suggest shifting plant invasion dynamics that decrease color diversity among flowers. This finding is consistent with prior work showing that plant invasions can decrease floral functional diversity, consequently decreasing the abundance of specialist pollinators, while increasing pollinator visitation to abundant, invasive species (Bartomeus et al. 2008; Kaiser‐Bunbury et al. 2017; Russo et al. 2019; Gaiarsa and Bascompte 2022). The observed shifts in color arise from shifts in floral species composition and abundance in response to nutrients (Midolo et al. 2019; Wilcots et al. 2021) rather than from direct within‐species effects of soil nutrients and chemistry on flower color (Grossenbacher et al. 2025).

Yellow and purple Asteraceae, which increased under nitrogen enrichment, played a core role in the plant–pollinator network by acting as a centralized hub that supported a diversity of generalized and specialized pollinators. Many of these Asteraceae species were invasive thistles, consistent with prior findings that abundant, highly visible invasive plant species can exert a strong influence on network structure (Bartomeus et al. 2008; Russo et al. 2019; Parra‐Tabla and Arceo‐Gómez 2021). Prior work has shown trait‐matching to be a critical determinant of plant–pollinator network structure (Watts et al. 2016; Peralta et al. 2020; Lautenschleger et al. 2021). These changes suggest functional shifts in pollination niches under global change (Phillips et al. 2020), with interactions between invasion and nutrient enrichment leading to subsequent effects for the composition and functioning of pollinator communities.

Theory suggests that differences in floral traits and their resulting niche differences can mediate how plant communities and their mutualisms with pollinators respond to anthropogenic global changes (Watts et al. 2016; Valdovinos et al. 2018). Empirical evidence for the utility of floral traits as an explanatory framework for categorizing pollinator niches and their responses to global change remains equivocal (Rosas‐Guerrero et al. 2014; Dellinger 2020; Lautenschleger et al. 2021; Hui et al. 2021). Plant functional traits can inform understandings of ecological processes such as community assembly (Funk et al. 2017). These findings suggest that changes in floral traits under nutrient enrichment may have implications for the provisioning of pollination services in grasslands (Maestre et al. 2022).

Our study is limited in that it does not measure additional traits relevant to pollinator attraction, such as floral scent, nectar quantity, and nectar quality (Baude et al. 2016; Burkle and Runyon 2017; Russo et al. 2023). Nutrient addition is shown to have both positive and negative effects on floral chemistry and reward quality of nectar and pollen (Burkle and Irwin 2010; Gijbels et al. 2015; Ceulemans et al. 2017; David et al. 2019; Russo et al. 2023), raising the need for future research. Furthermore, little is known as to whether eutrophication can alter floral microbe communities, which affect nectar chemistry and attractiveness and consequently pollination outcomes and pollinator nutrition (Pozo et al. 2020; Vannette 2020; Adler et al. 2021).

Our three grasslands varied in grazing history and spanned an aridity gradient. All the plant species found within the family Iridaceae were only present at Hopland, our wettest, most plant‐rich site, including *Sisyrinchium bellum* , a core hub within the plant–pollinator network at Hopland. The response of *Sisyrinchium bellum* at Hopland to nutrient effects strongly drove the response of Iridaceae and blue flowers to nutrients. This variation among grasslands raises questions for future research as to whether grazing history (Cutter et al. 2022; Ciavattini et al. 2023) and aridity (Forrest 2015; Thapa‐Magar et al. 2022) may further mediate how plant–pollinator interactions respond to nutrient enrichment.

Here, we demonstrate that two axes of pollinator niches, floral family and flower color, can explain shifts in floral composition under nutrient enrichment and their consequent effects on pollinator community composition. We further find that the outcomes of nutrient enrichment on plant–pollinator interactions vary by type of nutrient, with nitrogen having contrasting effects to those of other macro‐ and micro‐nutrients. Nitrogen addition increases yellow and purple flowers in Asteraceae, while other nutrients in the absence of nitrogen increase pink, blue, and green flowers in Geraniaceae, Fabaceae, and Euphorbiaceae. Critically, shifts in floral color and family may link eutrophication to shifts in pollinator community composition.

## Author Contributions

**Rebecca A. Nelson:** conceptualization (lead), data curation (lead), formal analysis (lead), funding acquisition (equal), investigation (lead), methodology (equal), project administration (lead), resources (lead), software (lead), supervision (lead), validation (lead), visualization (lead), writing – original draft (lead), writing – review and editing (lead). **Elizabeth T. Borer:** conceptualization (supporting), funding acquisition (equal), methodology (supporting), project administration (equal), resources (supporting), supervision (supporting), validation (supporting), visualization (supporting), writing – review and editing (supporting). **Eric W. Seabloom:** conceptualization (supporting), funding acquisition (equal), methodology (supporting), project administration (supporting), resources (supporting), supervision (supporting), validation (supporting), visualization (supporting), writing – review and editing (supporting).

## Conflicts of Interest

The authors declare no conflicts of interest.

## Supporting information


**Data S1:** Supporting Information.

## Data Availability

Data associated with this paper have been made publicly available through the Environmental Data Initiative. Data can be accessed and cited as follows: Nelson, R.A., E.W. Seabloom, and E.T. Borer. 2025. Data for “Floral color and family drive contrasting plant–pollinator responses to nutrient enrichment” by Rebecca A. Nelson, Elizabeth T. Borer, and Eric W. Seabloom 2025. Collected in California grasslands 2023 and 2024. ver 1. Environmental Data Initiative. https://doi.org/10.6073/pasta/049529d782e1fd6be82e9148df6b2e2a (Accessed 2025‐05‐07). Code associated with this paper and data can be accessed at: DOI https://doi.org/10.5281/zenodo.15603900.
