# Supplementary material for: Floral Color and Family Drive Contrasting Plant–Pollinator Responses to Nutrient Enrichment
Source: Ecol Evol. 2025 Sep 13;15(9):e72153. doi: 10.1002/ece3.72153 (PMC12432409; doi:10.1002/ece3.72153)
Supplement: Supplementary file 1 — Data S1: Supporting Information. [file ECE3-15-e72153-s001.docx]

**Supplemental Methods**

Pollinator visitation surveys at the Hopland Extension Center occurred on: 4/4/2023, 5/19/2023, 7/12/2023, 4/29/2024, 5/14/2024, and 7/16/2024. Pollinator visitation surveys at the Sierra Foothills Extension Center occurred on 4/18/2023-4/19/2023, 5/22/2023-5/23/2023, 6/7/2023-6/8/2023, 4/17/2024-4/18/2024, 5/20/2024-5/21/2024, and 6/27/2024.Pollinator visitation surveys at the UC McLaughlin Reserve occurred on 5/7/2023, 5/18/2023, 5/27/2023, 7/3/2023, 4/27/2024, 5/10/2024, and 7/9/2024. At the Hopland Extension Center, forb abundance surveys occurred on 4/25/2023, 5/3/2023, 5/19/2023, 7/12/2023, 4/29/2024, 5/14/2024, and 7/16/2024. At the Sierra Foothills Extension Center, forb abundance surveys occurred on 4/12/2023, 4/18/2023-4/19/2023, 5/22/2023-5/23/2023, 6/7/2023-6/8/2023, 4/17/2024-4/18/2023, 5/20/2024-5/21/2024, 6/27/2024. At the UC McLaughlin-Reserve, forb abundance surveys occurred on 4/13/2023, 4/29/2023, 5/18/2023, 5/27/2023, 7/4/2023, 4/26/2024, 5/7/2024, 5/10/2024, and 7/8/2024. Photographs of floral visitors associated with this project can be accessed at by filtering by date and location: <https://www.inaturalist.org/observations?subview=table&user_id=ranelson&verifiable=any&view=species>.

We further created plant-pollinator networks for each site, pooling data across all years, that examined interactions between pollinator morphospecies and plant family as well as pollinator morphospecies and flower color. In other words, the plant nodes in these networks represented unique plant taxonomic families or flower colors instead of unique plant species. We then generated two networks for each site, one network for plots where nitrogen was added (treatments N, NPKμ, NP, and NKμ) (hereafter referred to the nitrogen-enriched network) and one network for plots that did not have nitrogen added (treatments control, P, Kμ, and PKμ) (hereafter referred to as no-nitrogen network). We used nested contribution function in the “bipartite” package in R to calculate the individual contribution to network nestedness for each plant family and flower color for the nitrogen-enriched and no-nitrogen networks at each site. We used the following formula to compute two-tailed p-values for individual contribution to nestedness z-scores: p-value=2*(1−Φ(∣z∣)). Φ is the is the cumulative distribution function of a **standard normal distribution. A p value greater than the alpha level of 0.05 indicates that a plant flower color or family contributes more strongly to the nested structure of the network than the random null model used by the contribution to nestedness function** that controls for the effect of differences in network degree (number of interactions a given network node has).

To test for whether plant provenance (native vs exotic) further modulated changes in floral abundance under nutrient enrichment, we added a covariate of plant provenance to our negative binomial model of all fixed effects and their interactions (e.g. Floral_Abundance ~ Treatment*Color*Provenance + (1|Site/block)). We used Tukey HSD posthoc tests to examine how differences in floral abundance by provenance varied by each combination of flower color and treatment.

We further used the Jepson Manual (Baldwin & Goldman, 2012) and CalFlora.org to classify flower shape for reach forb species into the following categories “Composite", "Pea-like", "Salverform", "Rotate", "Funnel-shaped", "Spike", "Stellate", "Saccate", "Two-lipped", "Pilstillate", "Galeate" , and "Cruciform". We then fit a series of negative binomial models to test for the effects of treatment, flower shape, and their interaction on flower abundance and visitation as response variables (e.g. Floral_Abundance ~ Treatment+Flower Shape + Treatment xFlower Shape + (1|Site/block)). We included this as a supplementary analysis because flower shape was related to flower taxonomic family. For example, species in Asteraceae were always assigned a “Composite” flower shape, and species in Fabaceae were always assigned “Pea-like” flower shape.

**Supplemental Results**

Exotic purple flowers were more abundant than native purple flowers under potassium with micronutrient addition (z=2.211, p=0.0270), under nitrogen addition (z=2.073, p=0.0382), under combined nitrogen and potassium with micronutrients addition (z=2.894, p=0.0038), under combined nitrogen and phosphorus addition (z=3.589, p=0.0003), and under combined nitrogen, phosphorus and potassium with micronutrient addition (z=3.558, p=0.0004). Exotic yellow flowers were more abundant than native yellow flowers under combined nitrogen and phosphorus addition (z=5.050, p<.0001), combined nitrogen, phosphorus, and potassium with micronutrient addition (t=3.359, p=0.0008), phosphorus addition (z=2.402, p=0.0163), and combined phosphorus and potassium addition (z= 3.493, p=0.0005). Native pink flowers were more abundant than exotic pink flowers under combined nitrogen and potassium with micronutrients (z=-3.258, p=0.0011).

The abundance of composite-shaped flowers increased with nitrogen-addition relative to ambient conditions (Table S7), while the abundance of pea-shaped flowers decreased under combined nitrogen and phosphorus addition relative to ambient conditions (Table S7). Likewise, pistillate flowers, rotate (wheel-shaped), and stellate (star-shaped) flowers decreased in floral abundance with nitrogen addition relative to ambient conditions (Table S7). Salverform flowers increased in floral abundance with phosphorus addition relative to nitrogen-addition (Table S7).

Combined addition of nitrogen, phosphorus, and potassium with micronutrients decreased total pollinator visitation to salverform flowers relative to ambient conditions (z=-3.72, p=0.0037). For other flower shapes, total pollinator visitation did not significantly differ by nutrient treatment for a given flower shape.

**Supplemental Tables and Figures**


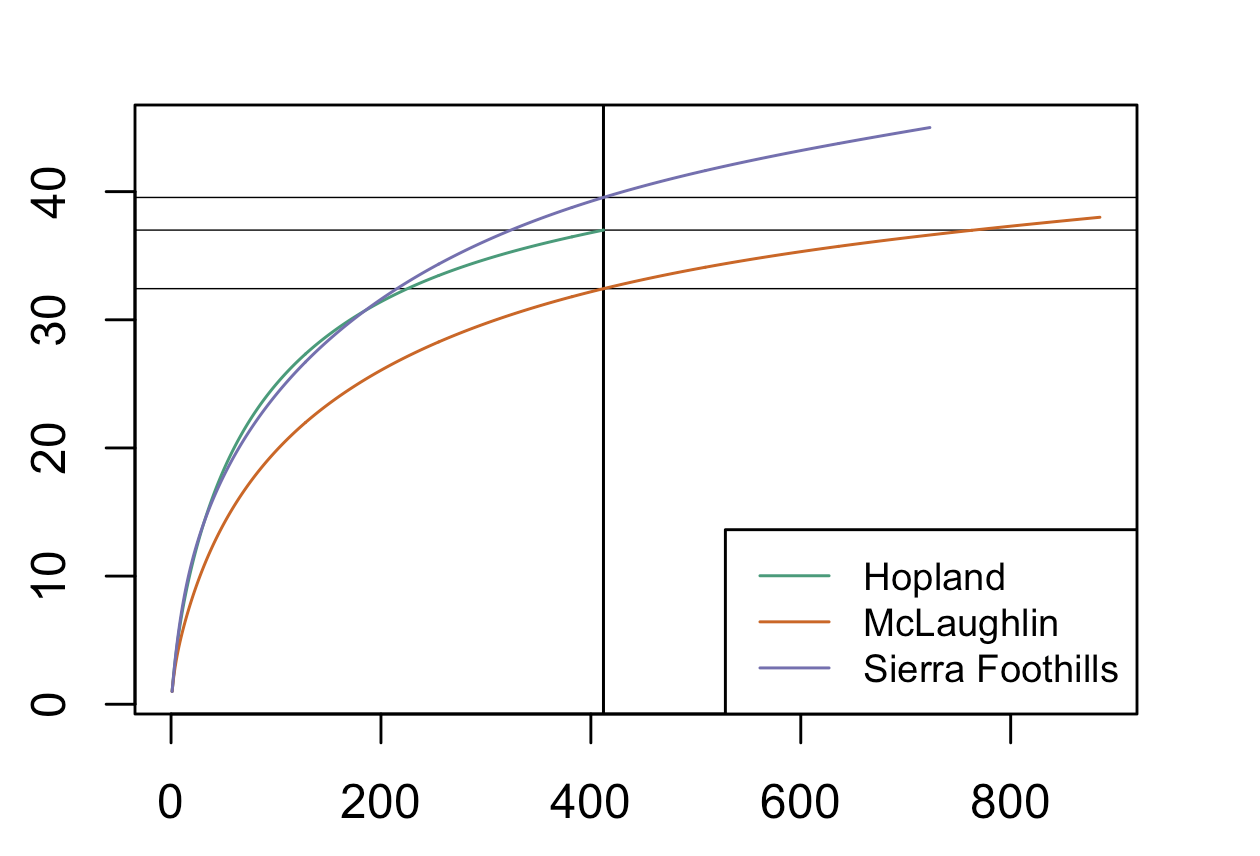


**Figure S1.** Species rarefaction curve by site for pollinator visitation data. Hopland is represented by teal, Sierra Foothills by purple, and McLaughlin by orange.


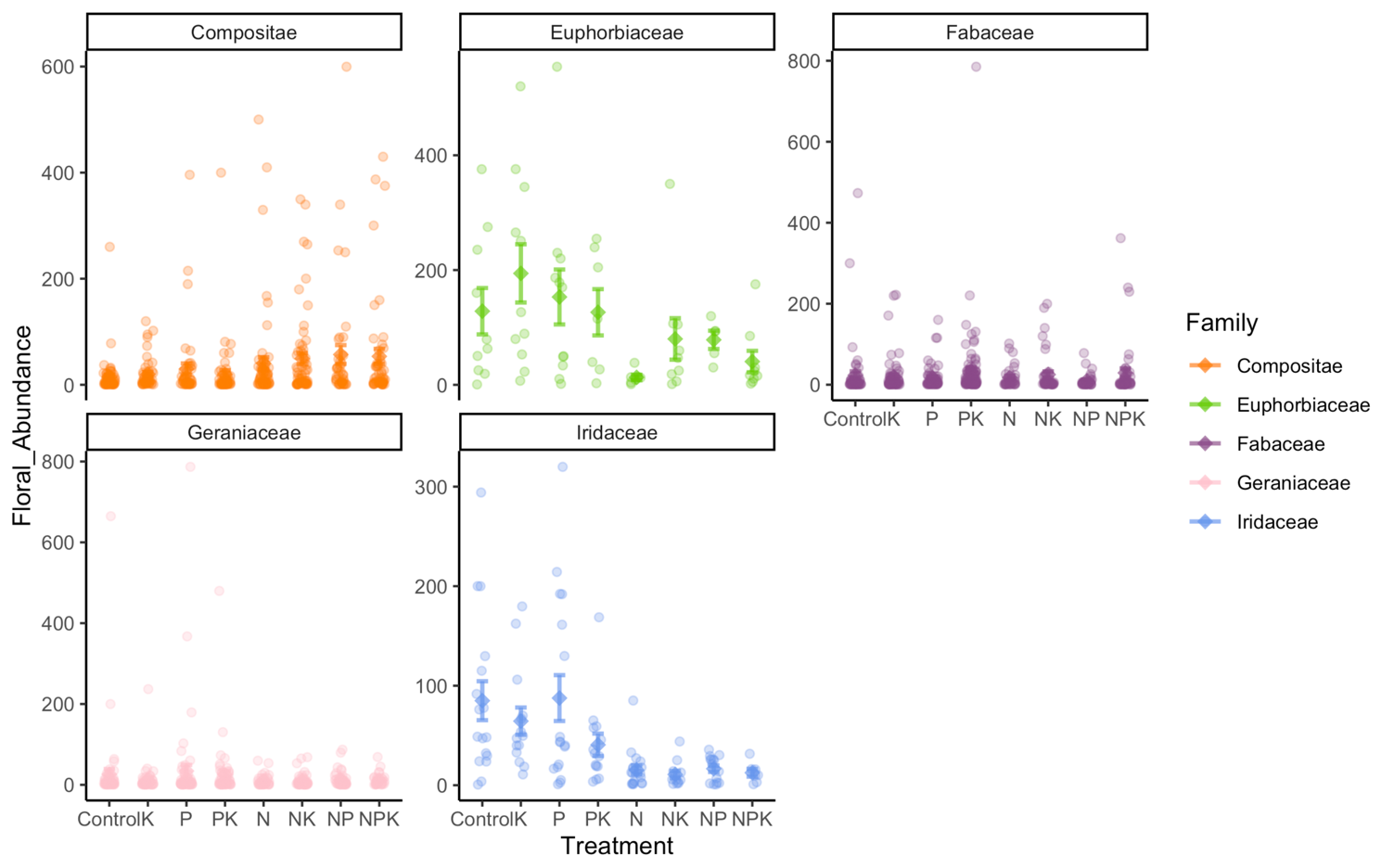


**Figure S2.** Floral abundance by treatment and floral family. Control refers to unfertilized control treatment, K refers to the addition of potassium with micronutrients, P refers to phosphorus addition, and N refers to nitrogen addition. Bars show mean with standard error. Each dot represents a unique combination of site and year.


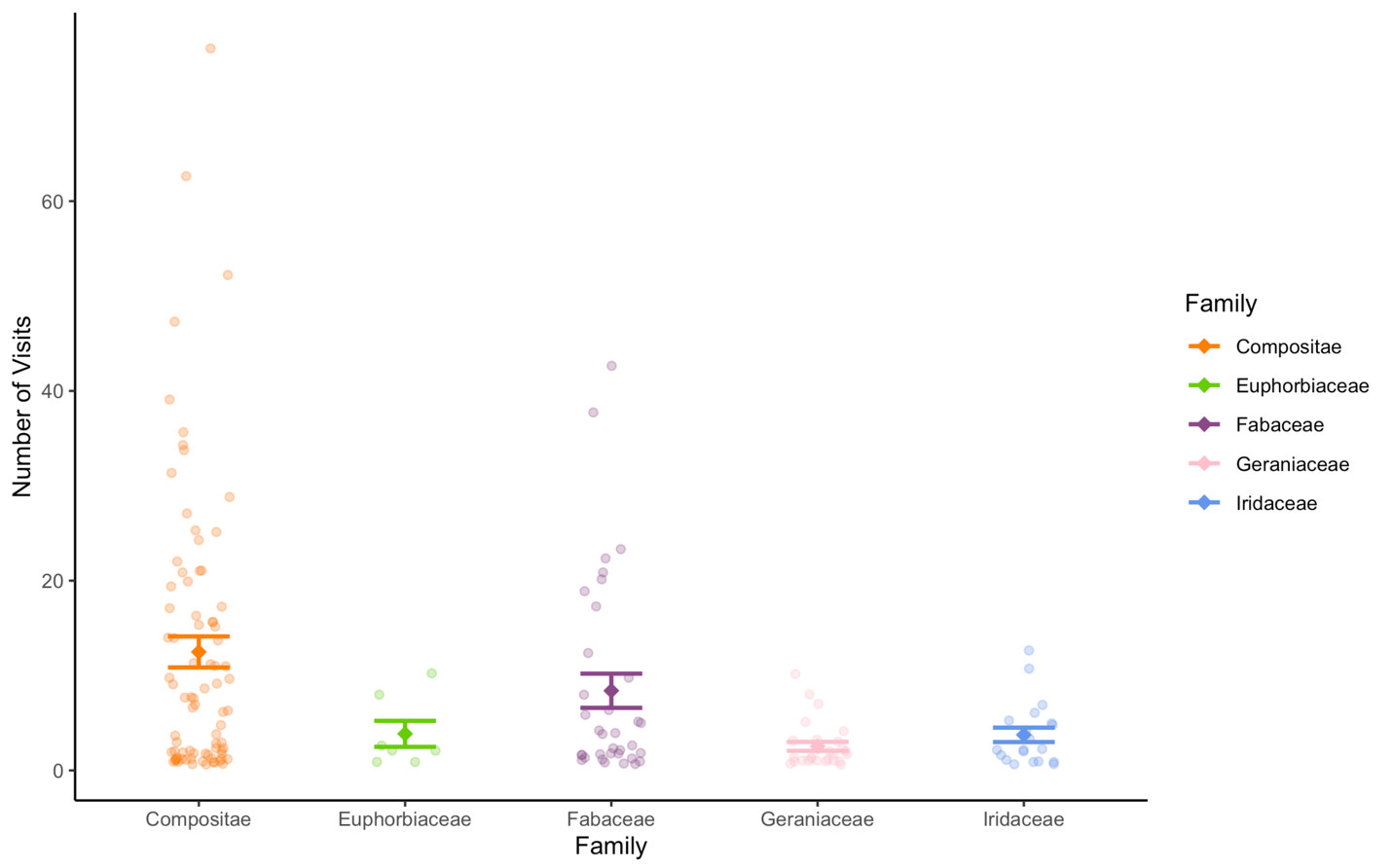


**Figure S3.** Number of pollinator visits by floral family. Bars show mean with standard error. Each dot represents a unique combination of site and year. Results were analyzed by fitting negative binomial mixed effects models.


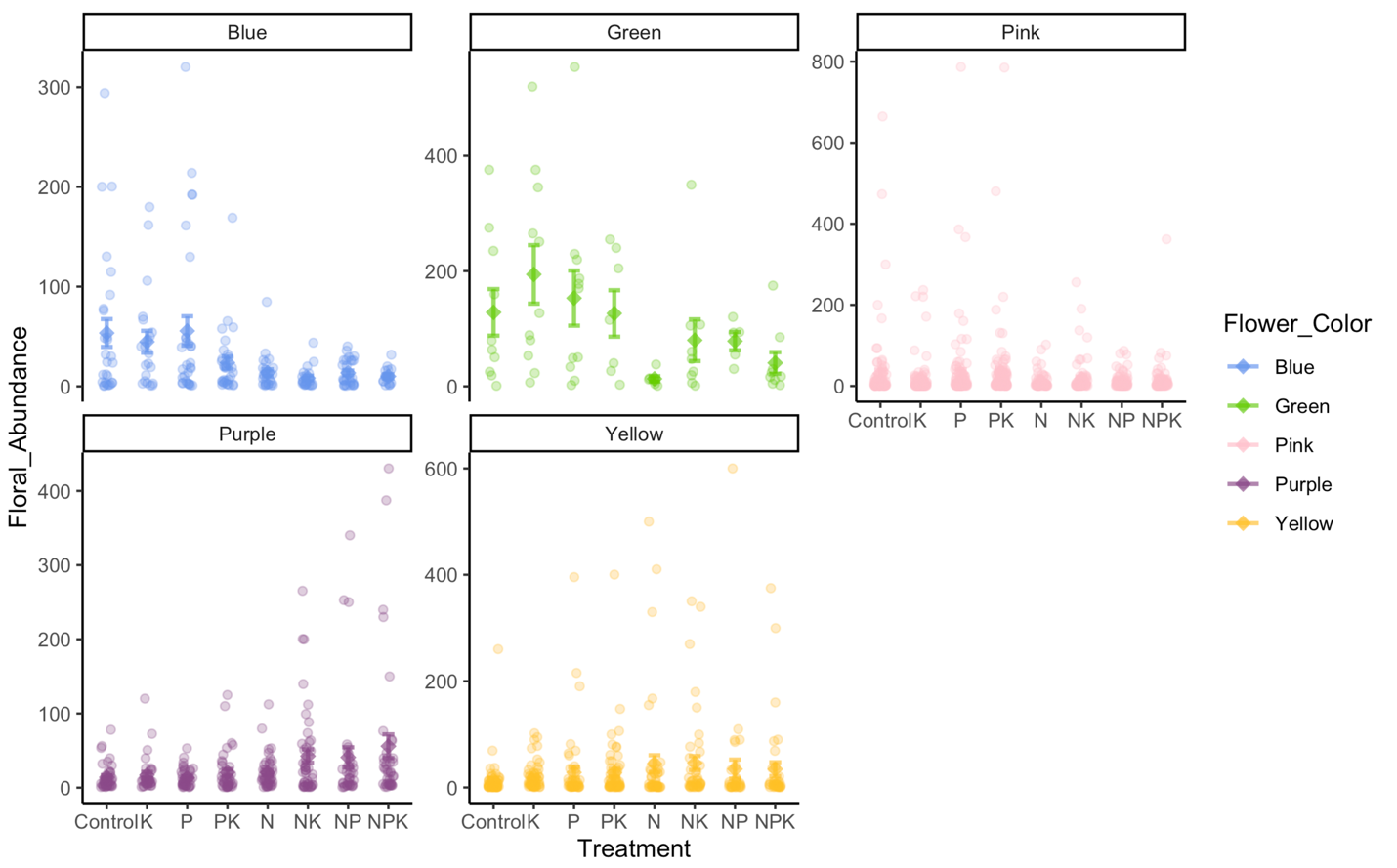


**Figure S4.** Floral abundance by treatment and floral color. Control refers to unfertilized control treatment, K refers to the addition of potassium with micronutrients, P refers to phosphorus addition, and N refers to nitrogen addition. Bars show mean with standard error. Each dot represents a unique combination of site and year.


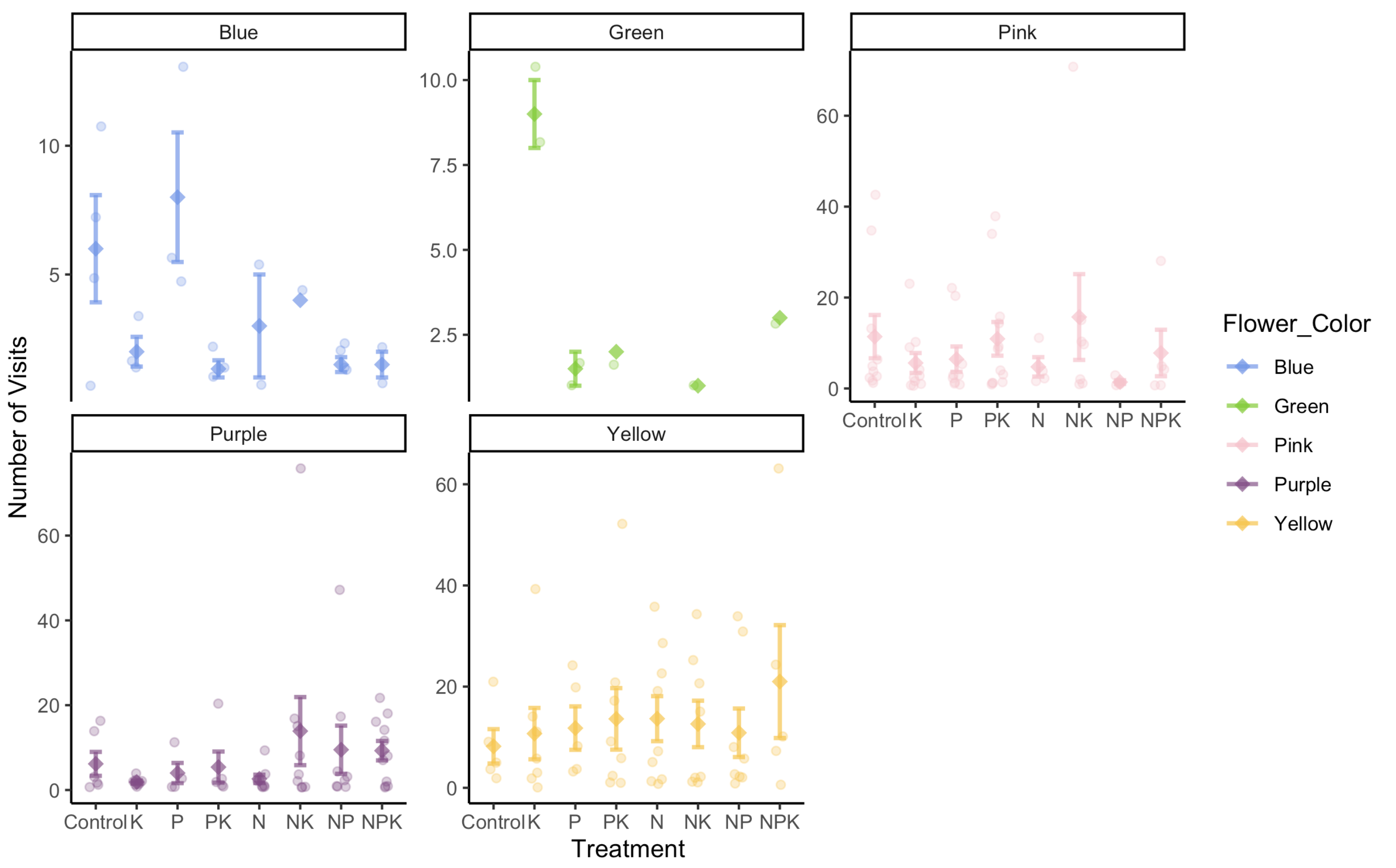


**Figure S5.** Number of pollinator visits (abundance) by treatment and floral color. Control refers to unfertilized control treatment, K refers to the addition of potassium with micronutrients, P refers to phosphorus addition, and N refers to nitrogen addition. Bars show mean with standard error. Each dot represents a unique combination of site and year.

**Table S1.** Plant Species List. The table lists the plant species (PLANT), its taxonomic family, flower color, and the sites at which this plant species was observed flowering within the experiment. The sites increase in aridity as follows: Hopland, McLaughlin, Sierra Foothills.

| **PLANT** | **Family** | **Flower_Color** | **Sites** |
| --- | --- | --- | --- |
| ***Achillea millefolium*** | Compositae | White | Hopland |
| ***Achyrachaena mollis*** | Compositae | Orange | McLaughlin |
| ***Acmispon americanus*** | Fabaceae | Pink | Hopland, Sierra_Foothills |
| ***Agoseris heterophylla*** | Compositae | Yellow | Sierra_Foothills, Hopland, McLaughlin |
| ***Amsinckia menziesii*** | Boraginaceae | Orange | Sierra_Foothills, Hopland |
| ***Anagallis arvensis*** | Primulaceae | Orange | Hopland, Sierra_Foothills |
| **Asteraceae sp.** | Compositae | NA | Hopland |
| **Brassicaceae sp.** | Brassicaceae | NA | Hopland |
| ***Brodiaea elegans*** | Asparagaceae | Purple | McLaughlin, Sierra_Foothills |
| ***Brodiaea sp.*** | Asparagaceae | Purple | Sierra_Foothills |
| ***Calandrinia menziesii*** | Montiaceae | Pink | McLaughlin, Hopland |
| ***Calochortus venutus*** | Liliaceae | White | McLaughlin |
| ***Calycadenia pauciflora*** | Compositae | White | McLaughlin |
| ***Cardamine oligosperma*** | Brassicaceae | White | Sierra_Foothills |
| ***Carduus pycnocephalus*** | Compositae | Purple | McLaughlin, Hopland, Sierra_Foothills |
| ***Castilleja attenuate*** | Scrophulariaceae | White | McLaughlin |
| ***Castilleja rubicundula*** | Orbobanchaceae | White | McLaughlin |
| ***Centaurea melitensis*** | Compositae | Yellow | Hopland |
| ***Centaurea solstitialis*** | Compositae | Yellow | McLaughlin, Hopland |
| ***Cerastium glomerium*** | Caryophyllaceae | White | Hopland |
| ***Chlorogalum pomeridianum*** | Hyacinthaceae | White | McLaughlin |
| ***Clarkia purpurea*** | Onagraceae | Pink | Sierra_Foothills, McLaughlin |
| ***Claytonia perfoliate*** | Montiaceae | White | Hopland |
| ***Convolvulus arvensis*** | Convolvulaceae | White | Sierra_Foothills |
| ***Cordylanthus pilosus*** | Orbobanchaceae | White | Sierra_Foothills |
| ***Delphinium hesperium*** | Ranunculaceae | Pink | McLaughlin |
| ***Dichelostemma capitatum*** | Asparagaceae | Purple | Hopland, Sierra_Foothills |
| ***Dichelostemma congestum*** | Asparagaceae | Purple | Hopland |
| ***Dichelostemma multiflorum*** | Asparagaceae | Purple | Sierra_Foothills |
| ***Erodium botrys*** | Geraniaceae | Pink | Hopland, Sierra_Foothills, McLaughlin |
| ***Erodium cicutarium*** | Geraniaceae | Pink | McLaughlin, Hopland, Sierra_Foothills |
| ***Erythranthe guttata*** | Phrymaceae | Orange | McLaughlin |
| ***Euphorbia spathulata*** | Euphorbiaceae | Green | McLaughlin, Sierra_Foothills |
| ***Geranium carolinianum*** | Geraniaceae | Pink | Sierra_Foothills |
| ***Geranium dissectum*** | Geraniaceae | Pink | Hopland, Sierra_Foothills, McLaughlin |
| ***Geranium molle*** | Geraniaceae | Pink | Sierra_Foothills, Hopland |
| ***Hemizonia congesta*** | Compositae | White | McLaughlin |
| ***Hypericum perforatum*** | Hypericaceae | Yellow | Sierra_Foothills |
| ***Hypochaeris glabra*** | Compositae | Yellow | Sierra_Foothills, Hopland, McLaughlin |
| ***Iris macrosiphon*** | Iridaceae | Indigo | Hopland |
| ***Lactuca serriola*** | Compositae | Yellow | Sierra_Foothills, Hopland |
| ***Lagophylla minor*** | Compositae | Yellow | Sierra_Foothills |
| ***Lasthenia californica*** | Compositae | Yellow | McLaughlin |
| ***Lepidium nitidum*** | Brassicaceae | White | Sierra_Foothills |
| ***Leptosiphon bicolor*** | Polemoniaceae | Pink | Sierra_Foothills, Hopland |
| ***Leptosiphon sp.*** | Polemoniaceae | NA | Hopland |
| ***Lomatium hooverii*** | Apiaceae | Red | Hopland |
| ***Lupinus bicolor*** | Fabaceae | Indigo | Hopland, McLaughlin, Sierra_Foothills |
| ***Lupinus microcarpus*** | Fabaceae | Pink | McLaughlin |
| ***Madia gracilis*** | Compositae | Yellow | Hopland, Sierra_Foothills |
| ***Madia sativa*** | Compositae | Yellow | Sierra_Foothills |
| ***Medicago polymorpha*** | Fabaceae | Yellow | McLaughlin |
| ***Micropus californicus*** | Compositae | White | Hopland |
| ***Minuartia douglasii*** | Caryophyllaceae | White | McLaughlin |
| ***Navarretia intertexta*** | Polemoniaceae | White | Sierra_Foothills |
| ***Navarretia pubescens*** | Polemoniaceae | Purple | Sierra_Foothills |
| ***Petrorhagia dubia*** | Caryophyllaceae | Pink | Hopland, Sierra_Foothills |
| ***Plagiobothrys nothofulvus*** | Boraginaceae | White | Hopland, Sierra_Foothills |
| ***Ranunculus californicus*** | Ranunculaceae | Yellow | Hopland, McLaughlin |
| ***Rhumex sp.*** | Polygonaceae | Red | McLaughlin |
| ***Rigiopappus leptocladus*** | Compositae | Yellow | Hopland |
| ***Sanicula bipinnata*** | Apiaceae | Yellow | Sierra_Foothills |
| ***Sanicula sp.*** | Apiaceae | Yellow | Sierra_Foothills |
| ***Senecio vulgaris*** | Compositae | Yellow | McLaughlin, Sierra_Foothills |
| ***Sherardia arvensis*** | Rubiaceae | White | Sierra_Foothills |
| ***Sidalcea sp.*** | Malvaceae | Pink | McLaughlin, Hopland |
| ***Sisyrinchium bellum*** | Iridaceae | Indigo | Hopland |
| ***Sonchus asper*** | Compositae | Yellow | Hopland, McLaughlin, Sierra_Foothills |
| ***Stellaria media*** | Caryophyllaceae | White | Sierra_Foothills |
| ***Taraxacum officinale*** | Compositae | Yellow | Sierra_Foothills, McLaughlin |
| ***Torilis arvensis*** | Apiaceae | White | Sierra_Foothills |
| ***Tragopogon dubius*** | Compositae | Yellow | McLaughlin |
| ***Trifolium albopurpureum*** | Fabaceae | Purple | Hopland |
| ***Trifolium bifidum*** | Fabaceae | Pink | Hopland, McLaughlin |
| ***Trifolium ciliolatum*** | Fabaceae | Pink | Hopland |
| ***Trifolium dubium*** | Fabaceae | Yellow | Sierra_Foothills, Hopland |
| ***Trifolium fucatum*** | Fabaceae | White | McLaughlin |
| ***Trifolium gracilentum*** | Fabaceae | Pink | Hopland |
| ***Trifolium hirtum*** | Fabaceae | Pink | Hopland, McLaughlin, Sierra_Foothills |
| ***Trifolium microdon*** | Fabaceae | White | Hopland |
| ***Trifolium nigrescens*** | Fabaceae | White | Sierra_Foothills |
| ***Triphysaria eriantha*** | Orobanchaceae | Yellow | McLaughlin |
| ***Triteleia hyacinthina*** | Asparagaceae | White | Sierra_Foothills |
| ***Triteleia laxa*** | Asparagaceae | Purple | Sierra_Foothills, Hopland |
| **UNK FORB SP.** | NA | NA | Sierra_Foothills |
| ***Vicia americana*** | Fabaceae | Pink | Hopland |
| ***Vicia sativa*** | Fabaceae | Pink | Sierra_Foothills, Hopland |
| ***Vicia villosa*** | Fabaceae | Purple | Hopland, McLaughlin, Sierra_Foothills |

**Table S2.** Pollinator morphospecies list. This table identifies pollinator morphospecies identified to the most specific taxon possible (ARTH), the functional group to which a given morphospecies has been assigned, and the sites at which a particular morphospecies was observed.

| **ARTH** | **Morphospecies description** | **Functional Group** | **Sites** |
| --- | --- | --- | --- |
| **Bees** | | | |
| ***Andrena dissona*** | *Andrena dissona* | Short-tongued_Bees | McLaughlin |
| ***Andrena plana*** | *Andrena plana* | Short-tongued_Bees | McLaughlin |
| ***Andrena sp.*** | Unknown *Andrena sp.* | Short-tongued_Bees | Hopland, McLaughlin, Sierra_Foothills |
| ***Anthophora californica*** | *Anthophora californica* | Long-tongued_Bees | Hopland |
| ***Anthophora sp.*** | *Anthophora platti* | Long-tongued_Bees | McLaughlin, Hopland |
| ***Apis mellifera*** | *Apis mellifera* | Long-tongued_Bees | Hopland, Sierra_Foothills, McLaughlin |
| ***Bombus californicus*** | *Bombus californicus* | Long-tongued_Bees | Sierra_Foothills, McLaughlin |
| ***Bombus crotchii*** | *Bombus crotchii* | Long-tongued_Bees | McLaughlin |
| ***Bombus melanopygus*** | *Bombus melanopygus* | Long-tongued_Bees | Hopland, Sierra_Foothills |
| ***Bombus sp.*** | Unidentified *Bombus sp.* | Long-tongued_Bees | Sierra_Foothills |
| ***Bombus vosnesenskii*** | *Bombus vosnesenskii* | Long-tongued_Bees | Hopland, McLaughlin, Sierra_Foothills |
| ***Brachysomida californica*** | *Brachysomida californica* | Short-tongued_Bees | Hopland |
| ***Diadasia nigrifrons*** | *Diadasia nigrifrons* | Long-tongued_Bees | McLaughlin |
| ***Dianthidium dubium*** | *Dianthidium dubium* | Short-tongued_Bees | Sierra_Foothills |
| ***Eucera actuosa*** | *Eucera actuosa* | Long-tongued_Bees | McLaughlin |
| ***Habropoda sp.*** | *Habropoda sp.* | Long-tongued_Bees | Sierra_Foothills |
| **Halictidae** | Halictidae sp. | Short-tongued_Bees | Sierra_Foothills, McLaughlin |
| ***Halictus ligatus*** | *Halictus ligatus* | Short-tongued_Bees | McLaughlin |
| ***Halictus tripartitus*** | *Halictus tripartitus* | Short-tongued_Bees | McLaughlin, Sierra_Foothills, Hopland |
| ***Lasioglossum incompletum*** | *Lasioglossum incompletum* | Short-tongued_Bees | McLaughlin, Hopland, Sierra_Foothills |
| **Megachile sp.** | *Megachile apicalis* | Short-tongued_Bees | McLaughlin, Hopland, Sierra_Foothills |
| ***Melissodes lupina*** | *Melissodes lupina* | Short-tongued_Bees | McLaughlin |
| ***Mellisodes sp.*** | Unknown *Mellisodes sp.* | Short-tongued_Bees | McLaughlin |
| ***Nomada edwardsii*** | *Nomada edwardsii* | Kleptoparasites | Sierra_Foothills |
| ***Nomada hesperia*** | *Nomada hesperia* | Kleptobees | McLaughlin |
| ***Osmia cara*** | *Large blue Osmia spp. (Osmia cara/Osmia atrocyanea/Osmia trevoris)* | Short-tongued_Bees | Hopland, Sierra_Foothills |
| ***Osmia gabrielis*** | *Osmia gabrielis* | Short-tongued_Bees | Hopland |
| ***Osmia sp.*** | Unknown shiny *Osmia sp.* | Short-tongued_Bees | Hopland, Sierra_Foothills |
| **blue_black_Osmia** | Blue black *Osmia* *spp.* (*Osmia cyanonitiens/Osmia colarodensis*) | Short-tongued_Bees | Sierra_Foothills, Hopland |
| **blue_green_Osmia** | *Osmia aglaia* | Short-tongued_Bees | Sierra_Foothills |
| ***Panurginus sp.*** | *Panurginus sp.* | Short-tongued_Bees | McLaughlin, Sierra_Foothills, Hopland |
| **Anthophila** | Other bee species that did not match any of the other morphospecies. | NA | Sierra_Foothills, Hopland, McLaughlin |
| **Flies** | | | |
| **Black fly 1 (BF1)** | Tiny black Dipteran | Flies | McLaughlin |
| **Bombyliidae** | Unknown Bombyliidae species, gray and fuzzy | Flies | McLaughlin, Sierra_Foothills, Hopland |
| ***Bombylius major*** | *Bombylius major* | Flies | Sierra_Foothills, Hopland |
| ***Eupeodes fumipennis*** | *Eupeodes fumipennis* | Flies | Hopland |
| **Muscidae** | Muscidae | Flies | Sierra_Foothills, McLaughlin |
| ***Scaeva affinis*** | *Scaeva affinis* | Flies | McLaughlin, Hopland |
| **Syrphidae** | Unknown Syrphidae species that did not match any existing morphospecies | Flies | McLaughlin, Sierra_Foothills, Hopland |
| **Tachinidae** | Tachinidae | Flies | Sierra_Foothills |
| **Calligrapher** | *Toxomerus occidentalis* | Flies | Sierra_Foothills, McLaughlin |
| **green_bottle_fly** | *Lucilia sp.* | Flies | Sierra_Foothills |
| **Diptera** | Other fly species that did not match any of the other morphospecies. | Flies | McLaughlin, Hopland, Sierra_Foothills |
| **Beetles** | | | |
| ***Anthrenus sp.*** | Unindentified *Anthrenus sp.* | Beetles | McLaughlin, Sierra_Foothills |
| ***Anthrenus verbasci*** | *Anthrenus verbasci* | Beetles | Hopland |
| **Buprestidae** | Buprestidae | Beetles | Sierra_Foothills, Hopland |
| **Cerambycidae** | Cerambycidae | Beetles | Hopland |
| ***Epicauta puncticollis*** | *Epicauta puncticollis* | Beetles | Sierra_Foothills |
| ***Hippodamia convergens*** | *Hippodamia convergens* | Beetles | Hopland, McLaughlin, Sierra_Foothills |
| ***Listrus sp.*** | Medium, dark gray *Listrus sp.* | Beetles | Sierra_Foothills, McLaughlin, Hopland |
| **beetle_1 (*Listrus sp*.)** | Small black *Listrus sp*. | Beetles | McLaughlin, Hopland, Sierra_Foothills |
| ***Melanthaxia sp.*** | *Melanthaxia sp.* | Beetles | Sierra_Foothills |
| **Mordellidae** | Mordellidae | Beetles | Hopland |
| **Trichoda ornatus** | *Trichodes ornatus* | Beetles | Hopland, Sierra_Foothills |
| **small_red_beetle** | Small red Coleoptera sp. | Beetles | McLaughlin |
| **Coleoptera** | Other beetle species that did not match any of the other morphospecies. | Beetles | McLaughlin, Sierra_Foothills, Hopland |
| **Butterflies and Moths (Lepidoptera)** | | | |
| ***Battus philenor*** | *Battus philenor* | Leps | Sierra_Foothills |
| ***Coenonympha tullia*** | *Coenonympha tullia* | Leps | Hopland, Sierra_Foothills |
| ***Colias eurytheme*** | *Colias eurytheme* | Leps | McLaughlin |
| ***Glaucopsyche lygdamus*** | *Glaucopsyche lygdamus* | Leps | Sierra_Foothills, Hopland |
| ***Heliothodes diminutivus*** | *Heliothodes diminutivus* | Leps | McLaughlin, Sierra_Foothills |
| ***Icaricia acmon*** | *Icaricia acmon* | Leps | Hopland |
| ***Junonia grisea*** | *Junonia grisea* | Leps | McLaughlin |
| ***Phycoides mytilla*** | *Phycoides mytilla* | Leps | Hopland, Sierra_Foothills |
| ***Schinia pulchripennis*** | *Schinia pulchripennis* | Leps | Sierra_Foothills |
| ***Vanessa virginiensis*** | *Vanessa virginiensis* | Leps | Sierra_Foothills |
| **small_red_moth** | Small red moth (Lepidoptera) sp. | Leps | Sierra_Foothills |
| **Lepidoptera** | Other Lepidoptera that did not match any of the other morphospecies. | Leps | Hopland |
| **Wasps** | | | |
| **big_black_wasp** | Large black *Sphex sp.* | Wasps | McLaughlin, Sierra_Foothills |
| **black_gold_paperwasp** | *Polistes aurifer* | Wasps | McLaughlin |
| **Lacewings** | | | |
| **Chrysopidae** | Chrysopidae | Lacewings | Sierra_Foothills |

**Table S3.** Floral abundance by each pairwise combination of fertilization treatment by floral family for Asteraceae, Fabaceae, Iridaceae, Euphorbiaceae and Geraniaceae. P-values significant at an alpha level of p<0.05 are bolded. The overall z distribution has infinite degrees of freedom.

| contrast | Family | estimate | SE | z.ratio | p.value |
| --- | --- | --- | --- | --- | --- |
| Control – K | Asteraceae | -0.523 | 0.256 | -2.041 | 0.454 |
| Control – N | Asteraceae | -1.119 | 0.236 | -4.734 | **0.0000601** |
| Control – NK | Asteraceae | -1.340 | 0.239 | -5.602 | **0.000000590** |
| Control – NP | Asteraceae | -1.306 | 0.263 | -4.963 | **0.0000191** |
| Control – NPK | Asteraceae | -1.266 | 0.248 | -5.107 | **0.00000903** |
| Control – P | Asteraceae | -0.711 | 0.263 | -2.701 | 0.122 |
| Control – PK | Asteraceae | -0.246 | 0.250 | -0.985 | 0.977 |
| K – N | Asteraceae | -0.596 | 0.248 | -2.403 | 0.240 |
| K - NK | Asteraceae | -0.817 | 0.249 | -3.286 | **0.0227** |
| K - NP | Asteraceae | -0.783 | 0.277 | -2.831 | 0.0875 |
| K – NPK | Asteraceae | -0.7430 | 0.263 | -2.829 | 0.0879 |
| K – P | Asteraceae | -0.188 | 0.274 | -0.685 | 0.997 |
| K - PK | Asteraceae | 0.277 | 0.263 | 1.0563 | 0.966 |
| N - NK | Asteraceae | -0.221 | 0.229 | -0.961 | 0.980 |
| N - NP | Asteraceae | -0.187 | 0.259 | -0.720 | 0.996 |
| N - NPK | Asteraceae | -0.147 | 0.243 | -0.605 | 0.999 |
| N - P | Asteraceae | 0.408 | 0.256 | 1.594 | 0.754 |
| N - PK | Asteraceae | 0.873 | 0.244 | 3.587 | **0.00805** |
| NK - NP | Asteraceae | 0.0339 | 0.260 | 0.130 | 1.000 |
| NK - NPK | Asteraceae | 0.0737 | 0.244 | 0.302 | 1.000 |
| NK - P | Asteraceae | 0.629 | 0.255 | 2.464 | 0.211 |
| NK - PK | Asteraceae | 1.094 | 0.245 | 4.460 | **0.000221** |
| NP - NPK | Asteraceae | 0.0397 | 0.268 | 0.148 | 1.000 |
| NP - P | Asteraceae | 0.595 | 0.283 | 2.101 | 0.414 |
| NP - PK | Asteraceae | 1.060 | 0.269 | 3.935 | **0.00212** |
| NPK - P | Asteraceae | 0.555 | 0.267 | 2.0827 | 0.426 |
| NPK - PK | Asteraceae | 1.020 | 0.256 | 3.982 | **0.00175** |
| P - PK | Asteraceae | 0.465 | 0.268 | 1.736 | 0.664 |
| Control - K | Euphorbiaceae | -0.467 | 0.557 | -0.838 | 0.990 |
| Control - N | Euphorbiaceae | 2.242 | 0.635 | 3.531 | **0.009808** |
| Control - NK | Euphorbiaceae | 0.4230 | 0.587 | 0.721 | 0.996 |
| Control - NP | Euphorbiaceae | 0.460 | 0.697 | 0.660 | 0.998 |
| Control - NPK | Euphorbiaceae | 1.092 | 0.587 | 1.859 | 0.579 |
| Control - P | Euphorbiaceae | -0.185 | 0.554 | -0.334 | 1.000 |
| Control - PK | Euphorbiaceae | -0.0518 | 0.630 | -0.0822 | 1.000 |
| K - N | Euphorbiaceae | 2.709 | 0.624 | 4.340 | **0.000380** |
| K - NK | Euphorbiaceae | 0.890 | 0.571 | 1.560 | 0.774 |
| K - NP | Euphorbiaceae | 0.927 | 0.685 | 1.353 | 0.878 |
| K - NPK | Euphorbiaceae | 1.560 | 0.572 | 2.728 | 0.114 |
| K - P | Euphorbiaceae | 0.282 | 0.5429 | 0.5192 | 1.000 |
| K - PK | Euphorbiaceae | 0.415 | 0.614 | 0.677 | 0.998 |
| N - NK | Euphorbiaceae | -1.819 | 0.652 | -2.792 | 0.0969 |
| N - NP | Euphorbiaceae | -1.782 | 0.752 | -2.371 | 0.256 |
| N - NPK | Euphorbiaceae | -1.150 | 0.651 | -1.767 | 0.643 |
| N - P | Euphorbiaceae | -2.427 | 0.623 | -3.893 | 0.00251 |
| N - PK | Euphorbiaceae | -2.294 | 0.690 | -3.325 | **0.0200** |
| NK - NP | Euphorbiaceae | 0.0370 | 0.709 | 0.0521 | 1.000 |
| NK - NPK | Euphorbiaceae | 0.669 | 0.600 | 1.115 | 0.9538 |
| NK - P | Euphorbiaceae | -0.608 | 0.573 | -1.06 | 0.965 |
| NK - PK | Euphorbiaceae | -0.475 | 0.640 | -0.742 | 0.996 |
| NP - NPK | Euphorbiaceae | 0.633 | 0.710 | 0.891 | 0.987 |
| NP - P | Euphorbiaceae | -0.645 | 0.685 | -0.941 | 0.982 |
| NP - PK | Euphorbiaceae | -0.512 | 0.745 | -0.6878 | 0.997 |
| NPK - P | Euphorbiaceae | -1.278 | 0.574 | -2.225 | 0.336 |
| NPK - PK | Euphorbiaceae | -1.1443 | 0.6413 | -1.784 | 0.6307 |
| P - PK | Euphorbiaceae | 0.133 | 0.617 | 0.216 | 1.000 |
| Control - K | Fabaceae | 0.225 | 0.233 | 0.966 | 0.979 |
| Control - N | Fabaceae | 0.440 | 0.2860 | 1.538 | 0.787 |
| Control - NK | Fabaceae | 0.161 | 0.265 | 0.608 | 0.999 |
| Control - NP | Fabaceae | 1.024 | 0.282 | 3.632 | **0.00682** |
| Control - NPK | Fabaceae | 0.004 | 0.263 | 0.01559 | 1.000 |
| Control - P | Fabaceae | 0.295 | 0.238 | 1.239 | 0.9202 |
| Control - PK | Fabaceae | -0.279 | 0.219 | -1.275 | 0.908 |
| K - N | Fabaceae | 0.215 | 0.278 | 0.774 | 0.994 |
| K - NK | Fabaceae | -0.063 | 0.256 | -0.248 | 1.000 |
| K - NP | Fabaceae | 0.799 | 0.273 | 2.922 | 0.0682 |
| K - NPK | Fabaceae | -0.221 | 0.253 | -0.871 | 0.989 |
| K - P | Fabaceae | 0.0703 | 0.229 | 0.307 | 1.000 |
| K - PK | Fabaceae | -0.504 | 0.208 | -2.418 | 0.233 |
| N - NK | Fabaceae | -0.279 | 0.303 | -0.919 | 0.984 |
| N - NP | Fabaceae | 0.584 | 0.319 | 1.831 | 0.598 |
| N - NPK | Fabaceae | -0.436 | 0.301 | -1.448 | 0.835 |
| N - P | Fabaceae | -0.145 | 0.284 | -0.512 | 1.000 |
| N - PK | Fabaceae | -0.719 | 0.266 | -2.701 | 0.1222 |
| NK - NP | Fabaceae | 0.863 | 0.298 | 2.894 | 0.074 |
| NK - NPK | Fabaceae | -0.1571 | 0.278 | -0.565 | 0.999 |
| NK - P | Fabaceae | 0.134 | 0.262 | 0.510 | 1.000 |
| NK - PK | Fabaceae | -0.440 | 0.242 | -1.82 | 0.605 |
| NP - NPK | Fabaceae | -1.020 | 0.296 | -3.444 | **0.0133** |
| NP - P | Fabaceae | -0.729 | 0.278 | -2.618 | 0.149 |
| NP - PK | Fabaceae | -1.303 | 0.2608 | -4.997 | **0.00001606** |
| NPK - P | Fabaceae | 0.291 | 0.260 | 1.119 | 0.953 |
| NPK - PK | Fabaceae | -0.283 | 0.239 | -1.183 | 0.937 |
| P - PK | Fabaceae | -0.574 | 0.214 | -2.672 | 0.131 |
| Control - K | Geraniaceae | 0.538 | 0.265 | 2.029 | 0.462 |
| Control - N | Geraniaceae | 0.826 | 0.272 | 3.034 | **0.0496** |
| Control - NK | Geraniaceae | 0.834 | 0.263 | 3.174 | **0.0323** |
| Control - NP | Geraniaceae | 0.682 | 0.264 | 2.586 | 0.161 |
| Control - NPK | Geraniaceae | 0.473 | 0.297 | 1.597 | 0.753 |
| Control - P | Geraniaceae | -0.271 | 0.245 | -1.104 | 0.956 |
| Control - PK | Geraniaceae | -0.0492 | 0.257 | -0.191 | 1.000 |
| K - N | Geraniaceae | 0.288 | 0.269 | 1.073 | 0.962 |
| K - NK | Geraniaceae | 0.296 | 0.258 | 1.146 | 0.946 |
| K - NP | Geraniaceae | 0.144 | 0.262 | 0.548 | 0.999 |
| K - NPK | Geraniaceae | -0.0648 | 0.295 | -0.220 | 1.000 |
| K - P | Geraniaceae | -0.809 | 0.258 | -3.132 | **0.0369** |
| K - PK | Geraniaceae | -0.587 | 0.260 | -2.264 | 0.314 |
| N - NK | Geraniaceae | 0.008 | 0.265 | 0.0302 | 1.000 |
| N - NP | Geraniaceae | -0.1445 | 0.2697 | -0.536 | 1.000 |
| N - NPK | Geraniaceae | -0.353 | 0.299 | -1.179 | 0.938 |
| N - P | Geraniaceae | -1.097 | 0.266 | -4.120 | **0.0009902** |
| N - PK | Geraniaceae | -0.876 | 0.268 | -3.271 | **0.0238** |
| NK - NP | Geraniaceae | -0.153 | 0.260 | -0.587 | 0.999 |
| NK - NPK | Geraniaceae | -0.361 | 0.291 | -1.239 | 0.920 |
| NK - P | Geraniaceae | -1.105 | 0.256 | -4.315 | **0.000424** |
| NK - PK | Geraniaceae | -0.884 | 0.257 | -3.433 | **0.0139** |
| NP - NPK | Geraniaceae | -0.208 | 0.296 | -0.704 | 0.997 |
| NP - P | Geraniaceae | -0.953 | 0.256 | -3.720 | **0.00491** |
| NP - PK | Geraniaceae | -0.731 | 0.257 | -2.844 | 0.0845 |
| NPK - P | Geraniaceae | -0.744 | 0.291 | -2.556 | 0.172 |
| NPK - PK | Geraniaceae | -0.523 | 0.293 | -1.781 | 0.633 |
| P - PK | Geraniaceae | 0.222 | 0.249 | 0.888 | 0.987 |
| Control - K | Iridaceae | 0.216 | 0.459 | 0.471 | 1.000 |
| Control - N | Iridaceae | 1.642 | 0.428 | 3.836 | **0.00314** |
| Control - NK | Iridaceae | 1.955 | 0.4904938 | 3.985 | **0.00173** |
| Control - NP | Iridaceae | 1.630 | 0.4558 | 3.577 | **0.00834** |
| Control - NPK | Iridaceae | 1.807 | 0.581 | 3.111 | **0.0393** |
| Control - P | Iridaceae | -0.0911 | 0.436 | -0.209 | 1.000 |
| Control - PK | Iridaceae | 0.684 | 0.460 | 1.488 | 0.814 |
| K - N | Iridaceae | 1.426 | 0.451 | 3.160 | **0.0338** |
| K - NK | Iridaceae | 1.738 | 0.511 | 3.405 | **0.0153** |
| K - NP | Iridaceae | 1.414 | 0.478 | 2.958 | 0.0616 |
| K - NPK | Iridaceae | 1.591 | 0.598 | 2.661 | 0.135 |
| K - P | Iridaceae | -0.307 | 0.459 | -0.670 | 0.998 |
| K - PK | Iridaceae | 0.468 | 0.481 | 0.972 | 0.978 |
| N - NK | Iridaceae | 0.313 | 0.481 | 0.650 | 0.998 |
| N - NP | Iridaceae | -0.01162528481 | 0.447 | -0.0260 | 1.000 |
| N - NPK | Iridaceae | 0.165 | 0.573 | 0.288 | 1.000 |
| N - P | Iridaceae | -1.733 | 0.427 | -4.055 | **0.00130** |
| N - PK | Iridaceae | -0.958 | 0.452 | -2.120 | 0.402 |
| NK - NP | Iridaceae | -0.324 | 0.503 | -0.644 | 0.998 |
| NK - NPK | Iridaceae | -0.148 | 0.618 | -0.239 | 1.000 |
| NK - P | Iridaceae | -2.046 | 0.489 | -4.187 | **0.000742** |
| NK - PK | Iridaceae | -1.270 | 0.511 | -2.487 | 0.201 |
| NP - NPK | Iridaceae | 0.176 | 0.593 | 0.297 | 1.000 |
| NP - P | Iridaceae | -1.721 | 0.455 | -3.784 | **0.00385** |
| NP - PK | Iridaceae | -0.946 | 0.478 | -1.978 | 0.497 |
| NPK - P | Iridaceae | -1.898 | 0.580 | -3.275 | **0.0236** |
| NPK - PK | Iridaceae | -1.123 | 0.598 | -1.877 | 0.567 |
| P - PK | Iridaceae | 0.775 | 0.460 | 1.687 | 0.696 |

**Table S4.** Floral abundance by each pairwise combination of fertilization treatment by flower color. P-values significant at an alpha level of p<0.05 are bolded. NA denotes if a color wasn’t present in a certain combination of treatments. The overall z distribution has infinite degrees of freedom.

| contrast | Flower_Color | estimate | SE | z.ratio | p.value | |  |
| --- | --- | --- | --- | --- | --- | --- | --- |
| Control - K | Green | -0.373 | 0.5857924 | -0.636694021 | 0.998 | |  |
| Control - N | Green | 2.230 | 0.667 | 3.343 | **0.019** | |  |
| Control - NK | Green | 0.537 | 0.617 | 0.870 | 0.989 | |  |
| Control - NP | Green | 0.517 | 0.732 | 0.706 | 0.997 | |  |
| Control - NPK | Green | 1.177 | 0.618 | 1.905 | 0.548 | |  |
| Control - P | Green | -0.165 | 0.583 | -0.283 | 1.000 | |  |
| Control - PK | Green | 0.0804 | 0.663 | 0.121 | 1.000 | |  |
| K - N | Green | 2.603 | 0.656 | 3.971 | **0.00183** | |  |
| K - NK | Green | 0.910 | 0.600 | 1.517 | 0.799 | |  |
| K - NP | Green | 0.890 | 0.721 | 1.235 | 0.922 | |  |
| K - NPK | Green | 1.550 | 0.601 | 2.577 | 0.164 | |  |
| K - P | Green | 0.208 | 0.571 | 0.364 | 1.000 | |  |
| K - PK | Green | 0.453 | 0.645 | 0.702 | 0.997 | |  |
| N - NK | Green | -1.693 | 0.684 | -2.475 | 0.206 | |  |
| N - NP | Green | -1.714 | 0.790 | -2.170 | 0.370 | |  |
| N - NPK | Green | -1.054 | 0.683 | -1.542 | 0.785 | |  |
| N - P | Green | -2.396 | 0.655 | -3.656 | 0.00624 | |  |
| N - PK | Green | -2.150 | 0.725 | -2.967 | 0.0602 | |  |
| NK - NP | Green | -0.0203 | 0.746 | -0.027246255 | 1.000 | |  |
| NK - NPK | Green | 0.640 | 0.631 | 1.013020003 | 0.973 | |  |
| NK - P | Green | -0.702 | 0.603 | -1.164595048 | 0.942 | |  |
| NK - PK | Green | -0.457 | 0.673 | -0.678532583 | 0.998 | |  |
| NP - NPK | Green | 0.660 | 0.747 | 0.883860487 | 0.988 | |  |
| NP - P | Green | -0.682 | 0.721 | -0.945817540 | 0.981 | |  |
| NP - PK | Green | -0.436 | 0.783 | -0.557061643 | 0.999 | |  |
| NPK - P | Green | -1.342 | 0.604 | -2.221930631 | 0.338 | |  |
| NPK - PK | Green | -1.096 | 0.674 | -1.625558385 | 0.735 | |  |
| P - PK | Green | 0.245 | 0.649 | 0.378011849 | 1.000 | |  |
| Control - K | Blue | 0.135 | 0.387 | 0.348743084 | 1.000 | |  |
| Control - N | Blue | 1.341 | 0.372 | 3.606519597 | **0.00750** | |  |
| Control - NK | Blue | 1.835 | 0.396 | 4.633033705 | **0.0000978** | |  |
| Control - NP | Blue | 1.429 | 0.362 | 3.942219965 | **0.00206** | |  |
| Control - NPK | Blue | 1.658 | 0.427 | 3.883609465 | **0.00261** | |  |
| Control - P | Blue | -0.058 | 0.352 | -0.165274962 | 1.000 | |  |
| Control - PK | Blue | 0.760 | 0.353 | 2.152971803 | 0.381 | |  |
| K - N | Blue | 1.206 | 0.400 | 3.018816458 | 0.0518 | |  |
| K - NK | Blue | 1.700 | 0.422 | 4.027240296 | **0.00146** | |  |
| K - NP | Blue | 1.294 | 0.392 | 3.303774436 | **0.0214** | |  |
| K - NPK | Blue | 1.523 | 0.451 | 3.375518239 | **0.0169** | |  |
| K - P | Blue | -0.193 | 0.381 | -0.506466546 | 1.000 | |  |
| K - PK | Blue | 0.625 | 0.382 | 1.635865823 | 0.728 | |  |
| N - NK | Blue | 0.493 | 0.406 | 1.214045431 | 0.928 | |  |
| N - NP | Blue | 0.087 | 0.375 | 0.232795576 | 1.000 | |  |
| N - NPK | Blue | 0.317 | 0.437 | 0.724874969 | 0.996 | |  |
| N - P | Blue | -1.400 | 0.366 | -3.828617512 | **0.00324** | |  |
| N - PK | Blue | -0.581 | 0.367 | -1.583 | 0.760 | |  |
| NK - NP | Blue | -0.406 | 0.397 | -1.023 | 0.971 | |  |
| NK - NPK | Blue | -0.176 | 0.456 | -0.387 | 1.000 | |  |
| NK - P | Blue | -1.893 | 0.389 | -4.865 | **0.0000314** | |  |
| NK - PK | Blue | -1.074 | 0.391 | -2.75 | 0.108 | |  |
| NP - NPK | Blue | 0.230 | 0.429 | 0.536 | 0.999 | |  |
| NP - P | Blue | -1.487 | 0.356 | -4.177 | **0.000774** | |  |
| NP - PK | Blue | -0.668 | 0.358 | -1.869 | 0.573 | |  |
| NPK - P | Blue | -1.717 | 0.421 | -4.08 | 0.00118 | |  |
| NPK - PK | Blue | -0.898 | 0.423 | -2.125 | 0.398 | |  |
| P - PK | Blue | 0.819 | 0.347 | 2.359 | 0.262 | |  |
| Control - K | Orange | -0.032 | 0.643 | -0.0504 | 1.000 | |  |
| Control - N | Orange | 0.857 | 0.570 | 1.504 | 0.806 | |  |
| Control - NK | Orange | 0.223 | 0.679 | 0.329 | 1.000 | |  |
| Control - NP | Orange | -0.0301 | 0.596 | -0.050 | 1.000 | |  |
| Control - NPK | Orange | -0.918 | 0.564 | -1.629 | 0.733 | |  |
| Control - P | Orange | -1.932 | 0.715 | -2.702 | 0.122 | |  |
| Control - PK | Orange | 0.801 | 0.625 | 1.282 | 0.906 | |  |
| K - N | Orange | 0.890 | 0.660 | 1.347 | 0.881 | |  |
| K - NK | Orange | 0.256 | 0.753 | 0.340 | 1.000 | |  |
| K - NP | Orange | 0.00236 | 0.679 | 0.00347 | 1.000 | |  |
| K - NPK | Orange | -0.886 | 0.6496 | -1.3635 | 0.874 | |  |
| K - P | Orange | -1.900 | 0.784 | -2.422 | 0.230 | |  |
| K - PK | Orange | 0.834 | 0.711 | 1.173 | 0.940 | |  |
| N - NK | Orange | -0.634 | 0.695 | -0.911 | 0.985 | |  |
| N - NP | Orange | -0.887 | 0.615 | -1.443 | 0.837 | |  |
| N - NPK | Orange | -1.776 | 0.583 | -3.043 | 0.0482 | |  |
| N - P | Orange | -2.789 | 0.731 | -3.815 | **0.0034** | |  |
| N - PK | Orange | -0.0558 | 0.640 | -0.0872 | 1.000 | |  |
| NK - NP | Orange | -0.253 | 0.714 | -0.355 | 1.000 | |  |
| NK - NPK | Orange | -1.142 | 0.686 | -1.664 | 0.711 | |  |
| NK - P | Orange | -2.156 | 0.815 | -2.646 | 0.1397 | |  |
| NK - PK | Orange | 0.578 | 0.743 | 0.778 | 0.994 | |  |
| NP - NPK | Orange | -0.888 | 0.604 | -1.471 | 0.823 | |  |
| NP - P | Orange | -1.902 | 0.747 | -2.547 | 0.176 | |  |
| NP - PK | Orange | 0.831 | 0.668 | 1.244 | 0.919 | |  |
| NPK - P | Orange | -1.014 | 0.720 | -1.408 | 0.8539 | |  |
| NPK - PK | Orange | 1.719 | 0.640 | 2.687 | 0.126 | |  |
| P - PK | Orange | 2.734 | 0.777 | 3.518 | **0.0103** | |  |
| Control - K | Pink | 0.493 | 0.198 | 2.49 | 0.198 | |  |
| Control - N | Pink | 0.922 | 0.216 | 4.272 | **0.000512** | |  |
| Control - NK | Pink | 0.529 | 0.212 | 2.496 | 0.197 | |  |
| Control - NP | Pink | 0.899 | 0.211 | 4.261 | **0.000540** | |  |
| Control - NPK | Pink | 0.565 | 0.219 | 2.580 | 0.163 | |  |
| Control - P | Pink | 0.0002 | 0.194 | 0.00107 | 1.000 | |  |
| Control - PK | Pink | -0.106 | 0.196 | -0.541 | 0.999 | |  |
| K - N | Pink | 0.429 | 0.212 | 2.027 | 0.464 | |  |
| K - NK | Pink | 0.0359 | 0.209 | 0.172 | 1.000 | |  |
| K - NP | Pink | 0.406 | 0.207 | 1.959 | 0.510 | |  |
| K - NPK | Pink | 0.0722 | 0.216 | 0.334 | 1.000 | |  |
| K - P | Pink | -0.493 | 0.195 | -2.525 | 0.185 | |  |
| K - PK | Pink | -0.599 | 0.193 | -3.097 | 0.0411 | |  |
| N - NK | Pink | -0.393 | 0.226 | -1.740 | 0.660 | |  |
| N - NP | Pink | -0.0236 | 0.224 | -0.105 | 1.000 | |  |
| N - NPK | Pink | -0.357 | 0.232 | -1.539 | 0.786 | |  |
| N - P | Pink | -0.922 | 0.215 | -4.298 | **0.000457** | |  |
| N - PK | Pink | -1.028 | 0.212 | -4.849 | **0.00003398** | |  |
| NK - NP | Pink | 0.370 | 0.220 | 1.678 | 0.7018 | |  |
| NK - NPK | Pink | 0.0363 | 0.229 | 0.158 | 1.000 | |  |
| NK - P | Pink | -0.529 | 0.208 | -2.543 | 0.178 | |  |
| NK - PK | Pink | -0.6347687383 | 0.208 | -3.058 | **0.0462** | |  |
| NP - NPK | Pink | -0.334 | 0.229 | -1.459 | 0.829 | |  |
| NP - P | Pink | -0.898 | 0.207 | -4.34 | **0.000382** | |  |
| NP - PK | Pink | -1.0047 | 0.205 | -4.901 | **0.0000262** | |  |
| NPK - P | Pink | -0.5648 | 0.217 | -2.597 | 0.157 | |  |
| NPK - PK | Pink | -0.671 | 0.216 | -3.114 | **0.0391** | |  |
| P - PK | Pink | -0.106 | 0.192 | -0.553 | 0.999 | |  |
| Control - K | Purple | -0.115 | 0.32 | -0.360 | 1.000 | |  |
| Control - N | Purple | -0.332 | 0.297 | -1.117 | 0.953 | |  |
| Control - NK | Purple | -0.995 | 0.296 | -3.357 | 0.0180 | |  |
| Control - NP | Purple | -1.008 | 0.316 | -3.195 | **0.0304** | |  |
| Control - NPK | Purple | -1.281 | 0.306 | -4.188 | **0.0007405** | |  |
| Control - P | Purple | 0.125 | 0.314 | 0.399 | 1.000 | |  |
| Control - PK | Purple | -0.236 | 0.308 | -0.766 | 0.995 | |  |
| K - N | Purple | -0.217 | 0.315 | -0.689 | 0.997 | |  |
| K - NK | Purple | -0.880 | 0.314 | -2.801 | 0.0946 | |  |
| K - NP | Purple | -0.893 | 0.332 | -2.690 | 0.125 | |  |
| K - NPK | Purple | -1.166 | 0.324 | -3.597 | **0.00776** | |  |
| K - P | Purple | 0.241 | 0.328 | 0.733 | 0.996 | |  |
| K - PK | Purple | -0.121 | 0.325 | -0.372 | 1.000 | |  |
| N - NK | Purple | -0.663 | 0.288 | -2.301 | 0.293 | |  |
| N - NP | Purple | -0.676 | 0.310 | -2.184 | 0.361 | |  |
| N - NPK | Purple | -0.949 | 0.298 | -3.187 | **0.0312** | |  |
| N - P | Purple | 0.458 | 0.308 | 1.484 | 0.817 | |  |
| N - PK | Purple | 0.096 | 0.301 | 0.319 | 1.000 | |  |
| NK - NP | Purple | -0.0129 | 0.307 | -0.0421 | 1.000 | |  |
| NK - NPK | Purple | -0.286 | 0.295 | -0.968 | 0.979 | |  |
| NK - P | Purple | 1.121 | 0.308 | 3.6404 | **0.00662** | |  |
| NK - PK | Purple | 0.759 | 0.299 | 2.538 | 0.179 | |  |
| NP - NPK | Purple | -0.273 | 0.315 | -0.864 | 0.989 | |  |
| NP - P | Purple | 1.134 | 0.326 | 3.476 | **0.0119** | |  |
| NP - PK | Purple | 0.772 | 0.319 | 2.417 | 0.233 | |  |
| NPK - P | Purple | 1.406 | 0.318 | 4.424 | **0.000259** | |  |
| NPK - PK | Purple | 1.044 | 0.308 | 3.394 | 0.01584927200 | |  |
| P - PK | Purple | -0.3616521846 | 0.3191358 | -1.133 | 0.94963461195 | |  |
| Control - K | Red | NA |  |  |  | |  |
| Control - N | Red | NA |  |  |  | |  |
| Control - NK | Red | NA |  |  |  | |  |
| Control - NP | Red | NA |  |  |  | |  |
| Control - NPK | Red | NA |  |  |  | |  |
| Control - P | Red | NA |  |  |  | |  |
| Control - PK | Red | NA |  |  |  | |  |
| K - N | Red | -1.0123717316 | 1.803 | -0.561 | 0.943 | |  |
| K - NK | Red | NA |  |  |  | |  |
| K - NP | Red | -0.964 | 1.713 | -0.563 | 0.943 | |  |
| K - NPK | Red | NA |  |  |  | |  |
| K - P | Red | NA |  |  |  | |  |
| K - PK | Red | -0.694 | 2.073 | -0.335 | 0.987 | |  |
| N - NK | Red | NA |  |  |  | |  |
| N - NP | Red | 0.0483 | 1.280 | 0.0377 | 1.000 | |  |
| N - NPK | Red | NA |  |  |  | |  |
| N - P | Red | NA |  |  |  | |  |
| N - PK | Red | 0.3186368275 | 1.732 | 0.184 | 0.998 | |  |
| NK - NP | Red | NA |  |  |  | |  |
| NK - NPK | Red | NA |  |  |  | |  |
| NK - P | Red | NA |  |  |  | |  |
| NK - PK | Red | NA |  |  |  | |  |
| NP - NPK | Red | NA |  |  |  | |  |
| NP - P | Red | NA |  |  |  | |  |
| NP - PK | Red | 0.270 | 1.639 | 0.165 | 0.998 | |  |
| NPK - P | Red | NA |  |  |  | |  |
| NPK - PK | Red | NA |  |  |  | |  |
| P - PK | Red | NA |  |  |  | |  |
| Control - K | White | 0.329 | 0.361 | 0.911 | 0.985 | |  |
| Control - N | White | 0.917 | 0.399 | 2.352 | 0.266 | |  |
| Control - NK | White | 0.789 | 0.391 | 2.018 | 0.470 | |  |
| Control - NP | White | 0.767 | 0.467 | 1.644 | 0.724 | |  |
| Control - NPK | White | 0.331 | 0.473 | 0.700 | 0.997 | |  |
| Control - P | White | 0.522 | 0.384 | 1.358 | 0.876 | |  |
| Control - PK | White | 0.853 | 0.383 | 2.227 | 0.336 | |  |
| K - N | White | 0.588 | 0.354 | 1.662 | 0.712 | |  |
| K - NK | White | 0.460 | 0.351 | 1.312 | 0.895 | |  |
| K - NP | White | 0.438 | 0.438 | 0.999 | | 0.975 | |
| K - NPK | White | 0.00175 | 0.439 | 0.004 | | 1.000 | |
| K - P | White | 0.193 | 0.346 | 0.558 | | 0.999 | |
| K - PK | White | 0.524 | 0.345 | 1.518 | | 0.798 | |
| N - NK | White | -0.128 | 0.381 | -0.336 | | 1.000 | |
| N - NP | White | -0.150 | 0.462 | -0.325 | | 1.000 | |
| N - NPK | White | -0.586 | 0.465 | -1.260 | | 0.913 | |
| N - P | White | -0.395 | 0.376 | -1.0512 | | 0.966 | |
| N - PK | White | -0.0636 | 0.376 | -0.169 | | 1.000 | |
| NK - NP | White | -0.0223 | 0.461 | -0.0483 | | 1.000 | |
| NK - NPK | White | -0.458 | 0.459 | -0.998 | | 0.975 | |
| NK - P | White | -0.267 | 0.374 | -0.714 | | 0.997 | |
| NK - PK | White | 0.064 | 0.374 | 0.172 | | 1.000 | |
| NP - NPK | White | -0.436 | 0.532 | -0.819 | | 0.992 | |
| NP - P | White | -0.245 | 0.457 | -0.535 | | 1.000 | |
| NP - PK | White | 0.087 | 0.456 | 0.190 | | 1.000 | |
| NPK - P | White | 0.191 | 0.458 | 0.417 | | 1.000 | |
| NPK - PK | White | 0.522 | 0.457 | 1.142 | | 0.948 | |
| P - PK | White | 0.331 | 0.368 | 0.900 | | 0.986 | |
| Control - K | Yellow | -0.501 | 0.2585 | -1.937 | | 0.525 | |
| Control - N | Yellow | -1.241 | 0.271 | -4.572 | | **0.000131** | |
| Control - NK | Yellow | -1.398 | 0.278 | -5.031 | | **0.0000135** | |
| Control - NP | Yellow | -0.767 | 0.299 | -2.563 | | 0.1695 | |
| Control - NPK | Yellow | -0.822 | 0.289 | -2.855 | | 0.0830 | |
| Control - P | Yellow | -0.740 | 0.278 | -2.665 | | 0.133 | |
| Control - PK | Yellow | -0.6921219491 | 0.2552811 | -2.711 | | 0.119 | |
| K - N | Yellow | -0.7405131851 | 0.2704632 | -2.738 | | 0.111 | |
| K - NK | Yellow | -0.897 | 0.275 | -3.262 | | **0.0245** | |
| K - NP | Yellow | -0.267 | 0.300 | -0.889 | | 0.987 | |
| K - NPK | Yellow | -0.321 | 0.289 | -1.109 | | 0.955 | |
| K - P | Yellow | -0.239 | 0.275 | -0.870 | | 0.987 | |
| K - PK | Yellow | -0.191 | 0.2524670 | -0.758 | | 0.995 | |
| N - NK | Yellow | -0.157 | 0.288 | -0.545 | | 0.999 | |
| N - NP | Yellow | 0.474 | 0.309 | 1.533 | | 0.790 | |
| N - NPK | Yellow | 0.419 | 0.299 | 1.403 | | 0.856 | |
| N - P | Yellow | 0.502 | 0.287 | 1.75 | | 0.655 | |
| N - PK | Yellow | 0.549 | 0.267 | 2.058 | | 0.443 | |
| NK - NP | Yellow | 0.631 | 0.317 | 1.992 | | 0.487 | |
| NK - NPK | Yellow | 0.576 | 0.304 | 1.893 | | 0.555 | |
| NK - P | Yellow | 0.658 | 0.288 | 2.289 | | 0.300 | |
| NK - PK | Yellow | 0.706 | 0.273 | 2.587 | | 0.161 | |
| NP - NPK | Yellow | -0.055 | 0.322 | -0.169 | | 1.000 | |
| NP - P | Yellow | 0.0276 | 0.313 | 0.088 | | 1.000 | |
| NP - PK | Yellow | 0.075 | 0.292 | 0.257 | | 1.000 | |
| NPK - P | Yellow | 0.082 | 0.296 | 0.277 | | 1.000 | |
| NPK - PK | Yellow | 0.130 | 0.284 | 0.456 | | 1.000 | |
| P - PK | Yellow | 0.047 | 0.271 | 0.175 | | 1.000 | |

**Table S5.** Network individual nested contribution for family.

| **Family** | **Site** | **Without N**  **z-score; p-value** | **With N**  **z-score; p-value** |
| --- | --- | --- | --- |
| Asteraceae | Mclaughlin | 0.716; 0.474 | **3.356; 7.899 x1**$\boldsymbol{0}^{\boldsymbol{-4}}$ |
| Asteraceae | Hopland | 0.278; 0.781 | 2.161; 0.0307 |
| Asteraceae | Sierra Foothills | **3.272; 0.00107** | **4.071; 4.674x1**$\boldsymbol{0}^{\boldsymbol{-5}}$ |
| Euphorbiaceae | McLaughlin | -1.565; 0.118 | -1.924; 0.0543 |
| Fabaceae | McLaughlin | -1.582; 0.114 | -1.910; 0.0561 |
| Fabaceae | Hopland | 0.703; 0.482 | 0.750; 0.454 |
| Fabaceae | Sierra Foothills | **2.206; 0.0274** | -0.488; 0.625 |
| Geraniaceae | McLaughlin | -0.877; 0.381 | -1.501; 0.133 |
| Geraniaceae | Hopland | -1.107; 0.268 | -1.316; 0.188 |
| Geraniaceae | Sierra Foothills | 0.693; 0.488 | -0.905; 0.366 |
| Iridaceae | Hopland | 1.506; 0.132 | 0.525; 0.600 |

**Table S6.** Network individual nested contribution for flower color.

| **Color** | **Site** | **Without N**  **z-score; p-value** | **With N**  **z-score; p-value** |
| --- | --- | --- | --- |
| Yellow | Mclaughlin | 1.583; 0.113 | **2.215; 0.0268** |
| Yellow | Hopland | -0.226; 0.821 | -0.648; 0.517 |
| Yellow | Sierra Foothills | 1.103; 0.270 | **2.660; 0.008** |
| Green | McLaughlin | -0.323; 0.747 | -1.411; 0.158 |
| Purple | McLaughlin | -1.205; 0.228 | -0.998 0.318 |
| Purple | Hopland | 0.668; 0.504 | 1.578; 0.115 |
| Purple | Sierra Foothills | 0.616; 0.538 | 1.947; 0.0515 |
| Pink | McLaughlin | 1.829; 0.0673 | 0.416; 0.678 |
| Pink | Hopland | 0.586; 0.558 | -0.173; 0.863 |
| Pink | Sierra Foothills | **2.136; 0.0327** | 0.340; 0.734 |
| Blue | Hopland | 1.641; 0.101 | -0.115; 0.908 |

Table S7. Floral abundance response to nutrient treatments by flower shape. Degrees of freedom are infinite for negative binomial models.

| contrast | Flower_Shape | estimate | SE | df | z.ratio | p.value |
| --- | --- | --- | --- | --- | --- | --- |
| Control - K | Composite | -0.48 | 0.26 | Inf | -1.82 | 0.61 |
| Control - N | Composite | -1.15 | 0.24 | Inf | -4.74 | 0.00 |
| Control - NK | Composite | -1.39 | 0.25 | Inf | -5.67 | 0.00 |
| Control - NP | Composite | -1.33 | 0.27 | Inf | -4.92 | 0.00 |
| Control - NPK | Composite | -1.30 | 0.25 | Inf | -5.13 | 0.00 |
| Control - P | Composite | -0.72 | 0.27 | Inf | -2.66 | 0.13 |
| Control - PK | Composite | -0.25 | 0.26 | Inf | -0.97 | 0.98 |
| K - N | Composite | -0.67 | 0.25 | Inf | -2.65 | 0.14 |
| K - NK | Composite | -0.91 | 0.25 | Inf | -3.60 | 0.01 |
| K - NP | Composite | -0.85 | 0.28 | Inf | -3.01 | 0.05 |
| K - NPK | Composite | -0.83 | 0.27 | Inf | -3.09 | 0.04 |
| K - P | Composite | -0.24 | 0.28 | Inf | -0.87 | 0.99 |
| K - PK | Composite | 0.23 | 0.27 | Inf | 0.85 | 0.99 |
| N - NK | Composite | -0.24 | 0.24 | Inf | -1.03 | 0.97 |
| N - NP | Composite | -0.18 | 0.27 | Inf | -0.67 | 1.00 |
| N - NPK | Composite | -0.15 | 0.25 | Inf | -0.62 | 1.00 |
| N - P | Composite | 0.43 | 0.26 | Inf | 1.63 | 0.73 |
| N - PK | Composite | 0.90 | 0.25 | Inf | 3.61 | 0.01 |
| NK - NP | Composite | 0.06 | 0.27 | Inf | 0.24 | 1.00 |
| NK - NPK | Composite | 0.09 | 0.25 | Inf | 0.35 | 1.00 |
| NK - P | Composite | 0.67 | 0.26 | Inf | 2.57 | 0.17 |
| NK - PK | Composite | 1.14 | 0.25 | Inf | 4.55 | 0.00 |
| NP - NPK | Composite | 0.02 | 0.28 | Inf | 0.08 | 1.00 |
| NP - P | Composite | 0.61 | 0.29 | Inf | 2.09 | 0.42 |
| NP - PK | Composite | 1.08 | 0.28 | Inf | 3.90 | 0.00 |
| NPK - P | Composite | 0.58 | 0.27 | Inf | 2.14 | 0.39 |
| NPK - PK | Composite | 1.06 | 0.26 | Inf | 4.02 | 0.00 |
| P - PK | Composite | 0.47 | 0.28 | Inf | 1.71 | 0.68 |
| Control - K | Cruciform | -2.25 | 1.32 | Inf | -1.70 | 0.69 |
| Control - N | Cruciform | 0.13 | 1.64 | Inf | 0.08 | 1.00 |
| Control - NK | Cruciform | -2.07 | 1.21 | Inf | -1.71 | 0.68 |
| Control - NP | Cruciform | -0.12 | 1.34 | Inf | -0.09 | 1.00 |
| Control - NPK | Cruciform | -1.84 | 1.32 | Inf | -1.39 | 0.86 |
| Control - P | Cruciform | -1.60 | 1.61 | Inf | -0.99 | 0.98 |
| Control - PK | Cruciform | -1.16 | 1.62 | Inf | -0.72 | 1.00 |
| K - N | Cruciform | 2.38 | 1.62 | Inf | 1.47 | 0.82 |
| K - NK | Cruciform | 0.18 | 1.19 | Inf | 0.15 | 1.00 |
| K - NP | Cruciform | 2.14 | 1.32 | Inf | 1.62 | 0.74 |
| K - NPK | Cruciform | 0.41 | 1.30 | Inf | 0.32 | 1.00 |
| K - P | Cruciform | 0.65 | 1.60 | Inf | 0.41 | 1.00 |
| K - PK | Cruciform | 1.09 | 1.60 | Inf | 0.68 | 1.00 |
| N - NK | Cruciform | -2.20 | 1.53 | Inf | -1.44 | 0.84 |
| N - NP | Cruciform | -0.25 | 1.63 | Inf | -0.15 | 1.00 |
| N - NPK | Cruciform | -1.97 | 1.62 | Inf | -1.21 | 0.93 |
| N - P | Cruciform | -1.73 | 1.87 | Inf | -0.93 | 0.98 |
| N - PK | Cruciform | -1.29 | 1.87 | Inf | -0.69 | 1.00 |
| NK - NP | Cruciform | 1.95 | 1.21 | Inf | 1.62 | 0.74 |
| NK - NPK | Cruciform | 0.23 | 1.19 | Inf | 0.19 | 1.00 |
| NK - P | Cruciform | 0.47 | 1.51 | Inf | 0.31 | 1.00 |
| NK - PK | Cruciform | 0.91 | 1.51 | Inf | 0.60 | 1.00 |
| NP - NPK | Cruciform | -1.72 | 1.32 | Inf | -1.31 | 0.90 |
| NP - P | Cruciform | -1.48 | 1.61 | Inf | -0.92 | 0.98 |
| NP - PK | Cruciform | -1.04 | 1.61 | Inf | -0.65 | 1.00 |
| NPK - P | Cruciform | 0.24 | 1.60 | Inf | 0.15 | 1.00 |
| NPK - PK | Cruciform | 0.68 | 1.60 | Inf | 0.42 | 1.00 |
| P - PK | Cruciform | 0.44 | 1.85 | Inf | 0.24 | 1.00 |
| Control - K | Funnel-shaped | 0.56 | 0.41 | Inf | 1.37 | 0.87 |
| Control - N | Funnel-shaped | 0.78 | 0.42 | Inf | 1.86 | 0.58 |
| Control - NK | Funnel-shaped | 0.49 | 0.54 | Inf | 0.91 | 0.98 |
| Control - NP | Funnel-shaped | 1.01 | 0.42 | Inf | 2.38 | 0.25 |
| Control - NPK | Funnel-shaped | 0.53 | 0.50 | Inf | 1.05 | 0.97 |
| Control - P | Funnel-shaped | 0.75 | 0.44 | Inf | 1.71 | 0.68 |
| Control - PK | Funnel-shaped | 0.85 | 0.44 | Inf | 1.93 | 0.53 |
| K - N | Funnel-shaped | 0.23 | 0.41 | Inf | 0.55 | 1.00 |
| K - NK | Funnel-shaped | -0.07 | 0.52 | Inf | -0.13 | 1.00 |
| K - NP | Funnel-shaped | 0.45 | 0.41 | Inf | 1.08 | 0.96 |
| K - NPK | Funnel-shaped | -0.03 | 0.49 | Inf | -0.06 | 1.00 |
| K - P | Funnel-shaped | 0.20 | 0.43 | Inf | 0.46 | 1.00 |
| K - PK | Funnel-shaped | 0.30 | 0.43 | Inf | 0.69 | 1.00 |
| N - NK | Funnel-shaped | -0.30 | 0.54 | Inf | -0.55 | 1.00 |
| N - NP | Funnel-shaped | 0.22 | 0.43 | Inf | 0.52 | 1.00 |
| N - NPK | Funnel-shaped | -0.25 | 0.50 | Inf | -0.50 | 1.00 |
| N - P | Funnel-shaped | -0.03 | 0.44 | Inf | -0.07 | 1.00 |
| N - PK | Funnel-shaped | 0.07 | 0.45 | Inf | 0.16 | 1.00 |
| NK - NP | Funnel-shaped | 0.52 | 0.54 | Inf | 0.96 | 0.98 |
| NK - NPK | Funnel-shaped | 0.04 | 0.59 | Inf | 0.07 | 1.00 |
| NK - P | Funnel-shaped | 0.26 | 0.54 | Inf | 0.49 | 1.00 |
| NK - PK | Funnel-shaped | 0.37 | 0.55 | Inf | 0.66 | 1.00 |
| NP - NPK | Funnel-shaped | -0.48 | 0.51 | Inf | -0.94 | 0.98 |
| NP - P | Funnel-shaped | -0.25 | 0.44 | Inf | -0.57 | 1.00 |
| NP - PK | Funnel-shaped | -0.15 | 0.45 | Inf | -0.34 | 1.00 |
| NPK - P | Funnel-shaped | 0.22 | 0.51 | Inf | 0.44 | 1.00 |
| NPK - PK | Funnel-shaped | 0.33 | 0.52 | Inf | 0.63 | 1.00 |
| P - PK | Funnel-shaped | 0.10 | 0.46 | Inf | 0.22 | 1.00 |
| Control - K | Galeate |  |  |  |  |  |
| Control - N | Galeate | 0.22 | 1.22 | Inf | 0.18 | 1.00 |
| Control - NK | Galeate | 0.54 | 1.94 | Inf | 0.28 | 1.00 |
| Control - NP | Galeate | 0.54 | 1.55 | Inf | 0.35 | 1.00 |
| Control - NPK | Galeate | 0.92 | 1.93 | Inf | 0.47 | 1.00 |
| Control - P | Galeate | -0.20 | 1.47 | Inf | -0.14 | 1.00 |
| Control - PK | Galeate |  |  |  |  |  |
| K - N | Galeate |  |  |  |  |  |
| K - NK | Galeate |  |  |  |  |  |
| K - NP | Galeate |  |  |  |  |  |
| K - NPK | Galeate |  |  |  |  |  |
| K - P | Galeate |  |  |  |  |  |
| K - PK | Galeate |  |  |  |  |  |
| N - NK | Galeate | 0.32 | 1.77 | Inf | 0.18 | 1.00 |
| N - NP | Galeate | 0.32 | 1.34 | Inf | 0.24 | 1.00 |
| N - NPK | Galeate | 0.69 | 1.77 | Inf | 0.39 | 1.00 |
| N - P | Galeate | -0.43 | 1.24 | Inf | -0.34 | 1.00 |
| N - PK | Galeate |  |  |  |  |  |
| NK - NP | Galeate | 0.00 | 2.01 | Inf | 0.00 | 1.00 |
| NK - NPK | Galeate | 0.38 | 2.32 | Inf | 0.16 | 1.00 |
| NK - P | Galeate | -0.74 | 1.95 | Inf | -0.38 | 1.00 |
| NK - PK | Galeate |  |  |  |  |  |
| NP - NPK | Galeate | 0.38 | 2.01 | Inf | 0.19 | 1.00 |
| NP - P | Galeate | -0.74 | 1.57 | Inf | -0.47 | 1.00 |
| NP - PK | Galeate |  |  |  |  |  |
| NPK - P | Galeate | -1.12 | 1.95 | Inf | -0.57 | 0.99 |
| NPK - PK | Galeate |  |  |  |  |  |
| P - PK | Galeate |  |  |  |  |  |
| Control - K | Pea-like | 0.21 | 0.24 | Inf | 0.88 | 0.99 |
| Control - N | Pea-like | 0.42 | 0.29 | Inf | 1.43 | 0.84 |
| Control - NK | Pea-like | 0.14 | 0.27 | Inf | 0.52 | 1.00 |
| Control - NP | Pea-like | 0.98 | 0.29 | Inf | 3.39 | 0.02 |
| Control - NPK | Pea-like | -0.02 | 0.27 | Inf | -0.06 | 1.00 |
| Control - P | Pea-like | 0.32 | 0.24 | Inf | 1.32 | 0.89 |
| Control - PK | Pea-like | -0.31 | 0.22 | Inf | -1.36 | 0.87 |
| K - N | Pea-like | 0.21 | 0.28 | Inf | 0.74 | 1.00 |
| K - NK | Pea-like | -0.07 | 0.26 | Inf | -0.26 | 1.00 |
| K - NP | Pea-like | 0.77 | 0.28 | Inf | 2.75 | 0.11 |
| K - NPK | Pea-like | -0.23 | 0.26 | Inf | -0.87 | 0.99 |
| K - P | Pea-like | 0.11 | 0.23 | Inf | 0.48 | 1.00 |
| K - PK | Pea-like | -0.51 | 0.21 | Inf | -2.41 | 0.24 |
| N - NK | Pea-like | -0.28 | 0.31 | Inf | -0.90 | 0.99 |
| N - NP | Pea-like | 0.56 | 0.33 | Inf | 1.71 | 0.68 |
| N - NPK | Pea-like | -0.44 | 0.31 | Inf | -1.41 | 0.85 |
| N - P | Pea-like | -0.10 | 0.29 | Inf | -0.34 | 1.00 |
| N - PK | Pea-like | -0.73 | 0.27 | Inf | -2.66 | 0.14 |
| NK - NP | Pea-like | 0.84 | 0.31 | Inf | 2.75 | 0.11 |
| NK - NPK | Pea-like | -0.16 | 0.29 | Inf | -0.55 | 1.00 |
| NK - P | Pea-like | 0.18 | 0.27 | Inf | 0.67 | 1.00 |
| NK - PK | Pea-like | -0.45 | 0.25 | Inf | -1.80 | 0.62 |
| NP - NPK | Pea-like | -1.00 | 0.30 | Inf | -3.28 | 0.02 |
| NP - P | Pea-like | -0.66 | 0.28 | Inf | -2.31 | 0.29 |
| NP - PK | Pea-like | -1.29 | 0.27 | Inf | -4.81 | 0.00 |
| NPK - P | Pea-like | 0.34 | 0.27 | Inf | 1.27 | 0.91 |
| NPK - PK | Pea-like | -0.29 | 0.25 | Inf | -1.18 | 0.94 |
| P - PK | Pea-like | -0.63 | 0.22 | Inf | -2.85 | 0.08 |
| Control - K | Pilstillate | -0.35 | 0.39 | Inf | -0.90 | 0.99 |
| Control - N | Pilstillate | 1.81 | 0.48 | Inf | 3.75 | 0.00 |
| Control - NK | Pilstillate | 0.38 | 0.44 | Inf | 0.84 | 0.99 |
| Control - NP | Pilstillate | 0.58 | 0.50 | Inf | 1.16 | 0.94 |
| Control - NPK | Pilstillate | 1.04 | 0.47 | Inf | 2.23 | 0.33 |
| Control - P | Pilstillate | -0.40 | 0.43 | Inf | -0.95 | 0.98 |
| Control - PK | Pilstillate | -0.09 | 0.45 | Inf | -0.20 | 1.00 |
| K - N | Pilstillate | 2.15 | 0.47 | Inf | 4.61 | 0.00 |
| K - NK | Pilstillate | 0.72 | 0.43 | Inf | 1.70 | 0.69 |
| K - NP | Pilstillate | 0.93 | 0.49 | Inf | 1.90 | 0.55 |
| K - NPK | Pilstillate | 1.39 | 0.45 | Inf | 3.10 | 0.04 |
| K - P | Pilstillate | -0.05 | 0.41 | Inf | -0.13 | 1.00 |
| K - PK | Pilstillate | 0.26 | 0.44 | Inf | 0.60 | 1.00 |
| N - NK | Pilstillate | -1.43 | 0.52 | Inf | -2.77 | 0.10 |
| N - NP | Pilstillate | -1.22 | 0.57 | Inf | -2.15 | 0.38 |
| N - NPK | Pilstillate | -0.77 | 0.53 | Inf | -1.43 | 0.84 |
| N - P | Pilstillate | -2.21 | 0.50 | Inf | -4.41 | 0.00 |
| N - PK | Pilstillate | -1.89 | 0.52 | Inf | -3.63 | 0.01 |
| NK - NP | Pilstillate | 0.21 | 0.53 | Inf | 0.39 | 1.00 |
| NK - NPK | Pilstillate | 0.66 | 0.50 | Inf | 1.34 | 0.88 |
| NK - P | Pilstillate | -0.78 | 0.46 | Inf | -1.68 | 0.70 |
| NK - PK | Pilstillate | -0.46 | 0.49 | Inf | -0.96 | 0.98 |
| NP - NPK | Pilstillate | 0.46 | 0.55 | Inf | 0.83 | 0.99 |
| NP - P | Pilstillate | -0.99 | 0.52 | Inf | -1.89 | 0.56 |
| NP - PK | Pilstillate | -0.67 | 0.54 | Inf | -1.24 | 0.92 |
| NPK - P | Pilstillate | -1.44 | 0.48 | Inf | -2.98 | 0.06 |
| NPK - PK | Pilstillate | -1.13 | 0.50 | Inf | -2.24 | 0.33 |
| P - PK | Pilstillate | 0.31 | 0.47 | Inf | 0.66 | 1.00 |
| Control - K | Rotate | 0.44 | 0.25 | Inf | 1.76 | 0.65 |
| Control - N | Rotate | 0.82 | 0.25 | Inf | 3.22 | 0.03 |
| Control - NK | Rotate | 0.72 | 0.25 | Inf | 2.89 | 0.07 |
| Control - NP | Rotate | 0.79 | 0.25 | Inf | 3.22 | 0.03 |
| Control - NPK | Rotate | 0.44 | 0.28 | Inf | 1.59 | 0.76 |
| Control - P | Rotate | -0.13 | 0.23 | Inf | -0.57 | 1.00 |
| Control - PK | Rotate | 0.03 | 0.25 | Inf | 0.10 | 1.00 |
| K - N | Rotate | 0.37 | 0.25 | Inf | 1.48 | 0.82 |
| K - NK | Rotate | 0.27 | 0.25 | Inf | 1.11 | 0.95 |
| K - NP | Rotate | 0.35 | 0.25 | Inf | 1.43 | 0.84 |
| K - NPK | Rotate | 0.00 | 0.28 | Inf | -0.01 | 1.00 |
| K - P | Rotate | -0.57 | 0.25 | Inf | -2.33 | 0.28 |
| K - PK | Rotate | -0.42 | 0.25 | Inf | -1.66 | 0.71 |
| N - NK | Rotate | -0.10 | 0.25 | Inf | -0.40 | 1.00 |
| N - NP | Rotate | -0.02 | 0.25 | Inf | -0.09 | 1.00 |
| N - NPK | Rotate | -0.38 | 0.28 | Inf | -1.36 | 0.88 |
| N - P | Rotate | -0.95 | 0.25 | Inf | -3.81 | 0.00 |
| N - PK | Rotate | -0.79 | 0.25 | Inf | -3.12 | 0.04 |
| NK - NP | Rotate | 0.08 | 0.24 | Inf | 0.32 | 1.00 |
| NK - NPK | Rotate | -0.28 | 0.27 | Inf | -1.01 | 0.97 |
| NK - P | Rotate | -0.85 | 0.24 | Inf | -3.49 | 0.01 |
| NK - PK | Rotate | -0.69 | 0.25 | Inf | -2.79 | 0.10 |
| NP - NPK | Rotate | -0.36 | 0.27 | Inf | -1.30 | 0.90 |
| NP - P | Rotate | -0.93 | 0.24 | Inf | -3.86 | 0.00 |
| NP - PK | Rotate | -0.77 | 0.24 | Inf | -3.15 | 0.03 |
| NPK - P | Rotate | -0.57 | 0.27 | Inf | -2.09 | 0.42 |
| NPK - PK | Rotate | -0.41 | 0.28 | Inf | -1.49 | 0.81 |
| P - PK | Rotate | 0.16 | 0.24 | Inf | 0.66 | 1.00 |
| Control - K | Saccate | -0.29 | 1.01 | Inf | -0.28 | 1.00 |
| Control - N | Saccate | 0.73 | 0.91 | Inf | 0.81 | 0.99 |
| Control - NK | Saccate | 1.39 | 1.76 | Inf | 0.79 | 0.99 |
| Control - NP | Saccate | 1.48 | 1.32 | Inf | 1.12 | 0.95 |
| Control - NPK | Saccate | 0.60 | 1.05 | Inf | 0.58 | 1.00 |
| Control - P | Saccate | -1.56 | 0.83 | Inf | -1.88 | 0.56 |
| Control - PK | Saccate | -0.28 | 0.84 | Inf | -0.34 | 1.00 |
| K - N | Saccate | 1.02 | 1.03 | Inf | 0.99 | 0.98 |
| K - NK | Saccate | 1.67 | 1.82 | Inf | 0.92 | 0.98 |
| K - NP | Saccate | 1.76 | 1.41 | Inf | 1.25 | 0.92 |
| K - NPK | Saccate | 0.89 | 1.16 | Inf | 0.77 | 0.99 |
| K - P | Saccate | -1.27 | 0.96 | Inf | -1.33 | 0.89 |
| K - PK | Saccate | 0.00 | 0.97 | Inf | 0.00 | 1.00 |
| N - NK | Saccate | 0.65 | 1.77 | Inf | 0.37 | 1.00 |
| N - NP | Saccate | 0.74 | 1.34 | Inf | 0.55 | 1.00 |
| N - NPK | Saccate | -0.13 | 1.08 | Inf | -0.12 | 1.00 |
| N - P | Saccate | -2.29 | 0.85 | Inf | -2.69 | 0.13 |
| N - PK | Saccate | -1.02 | 0.87 | Inf | -1.17 | 0.94 |
| NK - NP | Saccate | 0.09 | 2.01 | Inf | 0.04 | 1.00 |
| NK - NPK | Saccate | -0.78 | 1.85 | Inf | -0.42 | 1.00 |
| NK - P | Saccate | -2.95 | 1.73 | Inf | -1.71 | 0.68 |
| NK - PK | Saccate | -1.67 | 1.74 | Inf | -0.96 | 0.98 |
| NP - NPK | Saccate | -0.87 | 1.44 | Inf | -0.61 | 1.00 |
| NP - P | Saccate | -3.03 | 1.28 | Inf | -2.37 | 0.26 |
| NP - PK | Saccate | -1.76 | 1.29 | Inf | -1.36 | 0.87 |
| NPK - P | Saccate | -2.16 | 1.00 | Inf | -2.15 | 0.38 |
| NPK - PK | Saccate | -0.89 | 1.01 | Inf | -0.88 | 0.99 |
| P - PK | Saccate | 1.28 | 0.78 | Inf | 1.63 | 0.73 |
| Control - K | Salverform | 0.40 | 0.47 | Inf | 0.85 | 0.99 |
| Control - N | Salverform | 0.74 | 0.43 | Inf | 1.74 | 0.66 |
| Control - NK | Salverform | 0.47 | 0.46 | Inf | 1.03 | 0.97 |
| Control - NP | Salverform | 0.19 | 0.46 | Inf | 0.40 | 1.00 |
| Control - NPK | Salverform | -0.42 | 0.46 | Inf | -0.91 | 0.99 |
| Control - P | Salverform | -0.75 | 0.46 | Inf | -1.63 | 0.73 |
| Control - PK | Salverform | 0.46 | 0.49 | Inf | 0.95 | 0.98 |
| K - N | Salverform | 0.34 | 0.44 | Inf | 0.77 | 0.99 |
| K - NK | Salverform | 0.07 | 0.47 | Inf | 0.15 | 1.00 |
| K - NP | Salverform | -0.22 | 0.47 | Inf | -0.47 | 1.00 |
| K - NPK | Salverform | -0.82 | 0.47 | Inf | -1.76 | 0.65 |
| K - P | Salverform | -1.16 | 0.47 | Inf | -2.48 | 0.21 |
| K - PK | Salverform | 0.06 | 0.50 | Inf | 0.11 | 1.00 |
| N - NK | Salverform | -0.27 | 0.42 | Inf | -0.64 | 1.00 |
| N - NP | Salverform | -0.56 | 0.42 | Inf | -1.33 | 0.89 |
| N - NPK | Salverform | -1.16 | 0.43 | Inf | -2.71 | 0.12 |
| N - P | Salverform | -1.49 | 0.43 | Inf | -3.50 | 0.01 |
| N - PK | Salverform | -0.28 | 0.44 | Inf | -0.63 | 1.00 |
| NK - NP | Salverform | -0.29 | 0.45 | Inf | -0.63 | 1.00 |
| NK - NPK | Salverform | -0.89 | 0.46 | Inf | -1.95 | 0.52 |
| NK - P | Salverform | -1.23 | 0.46 | Inf | -2.68 | 0.13 |
| NK - PK | Salverform | -0.01 | 0.48 | Inf | -0.03 | 1.00 |
| NP - NPK | Salverform | -0.61 | 0.45 | Inf | -1.33 | 0.89 |
| NP - P | Salverform | -0.94 | 0.45 | Inf | -2.07 | 0.43 |
| NP - PK | Salverform | 0.27 | 0.48 | Inf | 0.57 | 1.00 |
| NPK - P | Salverform | -0.33 | 0.45 | Inf | -0.74 | 1.00 |
| NPK - PK | Salverform | 0.88 | 0.49 | Inf | 1.80 | 0.62 |
| P - PK | Salverform | 1.21 | 0.49 | Inf | 2.49 | 0.20 |
| Control - K | Spike | -0.30 | 1.87 | Inf | -0.16 | 1.00 |
| Control - N | Spike |  |  |  |  |  |
| Control - NK | Spike |  |  |  |  |  |
| Control - NP | Spike | -2.59 | 2.12 | Inf | -1.22 | 0.74 |
| Control - NPK | Spike |  |  |  |  |  |
| Control - P | Spike | -1.12 | 1.93 | Inf | -0.58 | 0.98 |
| Control - PK | Spike | -0.02 | 2.32 | Inf | -0.01 | 1.00 |
| K - N | Spike |  |  |  |  |  |
| K - NK | Spike |  |  |  |  |  |
| K - NP | Spike | -2.29 | 1.61 | Inf | -1.42 | 0.61 |
| K - NPK | Spike |  |  |  |  |  |
| K - P | Spike | -0.82 | 1.35 | Inf | -0.60 | 0.97 |
| K - PK | Spike | 0.28 | 1.87 | Inf | 0.15 | 1.00 |
| N - NK | Spike |  |  |  |  |  |
| N - NP | Spike |  |  |  |  |  |
| N - NPK | Spike |  |  |  |  |  |
| N - P | Spike |  |  |  |  |  |
| N - PK | Spike |  |  |  |  |  |
| NK - NP | Spike |  |  |  |  |  |
| NK - NPK | Spike |  |  |  |  |  |
| NK - P | Spike |  |  |  |  |  |
| NK - PK | Spike |  |  |  |  |  |
| NP - NPK | Spike |  |  |  |  |  |
| NP - P | Spike | 1.47 | 1.67 | Inf | 0.88 | 0.90 |
| NP - PK | Spike | 2.57 | 2.11 | Inf | 1.22 | 0.74 |
| NPK - P | Spike |  |  |  |  |  |
| NPK - PK | Spike |  |  |  |  |  |
| P - PK | Spike | 1.10 | 1.92 | Inf | 0.57 | 0.98 |
| Control - K | Stellate | 0.34 | 0.48 | Inf | 0.70 | 1.00 |
| Control - N | Stellate | 1.49 | 0.49 | Inf | 3.06 | 0.05 |
| Control - NK | Stellate | 2.08 | 0.52 | Inf | 4.04 | 0.00 |
| Control - NP | Stellate | 1.66 | 0.50 | Inf | 3.32 | 0.02 |
| Control - NPK | Stellate | 1.81 | 0.64 | Inf | 2.84 | 0.09 |
| Control - P | Stellate | -0.08 | 0.48 | Inf | -0.17 | 1.00 |
| Control - PK | Stellate | 0.81 | 0.49 | Inf | 1.67 | 0.71 |
| K - N | Stellate | 1.15 | 0.50 | Inf | 2.33 | 0.28 |
| K - NK | Stellate | 1.74 | 0.52 | Inf | 3.34 | 0.02 |
| K - NP | Stellate | 1.32 | 0.51 | Inf | 2.60 | 0.15 |
| K - NPK | Stellate | 1.47 | 0.64 | Inf | 2.29 | 0.30 |
| K - P | Stellate | -0.42 | 0.48 | Inf | -0.86 | 0.99 |
| K - PK | Stellate | 0.47 | 0.49 | Inf | 0.96 | 0.98 |
| N - NK | Stellate | 0.59 | 0.52 | Inf | 1.12 | 0.95 |
| N - NP | Stellate | 0.17 | 0.51 | Inf | 0.33 | 1.00 |
| N - NPK | Stellate | 0.32 | 0.65 | Inf | 0.49 | 1.00 |
| N - P | Stellate | -1.57 | 0.49 | Inf | -3.23 | 0.03 |
| N - PK | Stellate | -0.68 | 0.50 | Inf | -1.38 | 0.87 |
| NK - NP | Stellate | -0.42 | 0.53 | Inf | -0.79 | 0.99 |
| NK - NPK | Stellate | -0.27 | 0.66 | Inf | -0.41 | 1.00 |
| NK - P | Stellate | -2.16 | 0.51 | Inf | -4.21 | 0.00 |
| NK - PK | Stellate | -1.27 | 0.52 | Inf | -2.43 | 0.23 |
| NP - NPK | Stellate | 0.15 | 0.65 | Inf | 0.23 | 1.00 |
| NP - P | Stellate | -1.74 | 0.50 | Inf | -3.49 | 0.01 |
| NP - PK | Stellate | -0.85 | 0.51 | Inf | -1.67 | 0.71 |
| NPK - P | Stellate | -1.89 | 0.64 | Inf | -2.97 | 0.06 |
| NPK - PK | Stellate | -1.00 | 0.65 | Inf | -1.55 | 0.78 |
| P - PK | Stellate | 0.89 | 0.49 | Inf | 1.83 | 0.60 |
| Control - K | Two-lipped | 0.57 | 0.77 | Inf | 0.75 | 1.00 |
| Control - N | Two-lipped | 1.19 | 1.10 | Inf | 1.07 | 0.96 |
| Control - NK | Two-lipped | -0.90 | 0.96 | Inf | -0.94 | 0.98 |
| Control - NP | Two-lipped | 1.06 | 0.96 | Inf | 1.11 | 0.96 |
| Control - NPK | Two-lipped | 0.81 | 0.83 | Inf | 0.98 | 0.98 |
| Control - P | Two-lipped | -0.18 | 0.75 | Inf | -0.24 | 1.00 |
| Control - PK | Two-lipped | -0.05 | 0.77 | Inf | -0.06 | 1.00 |
| K - N | Two-lipped | 0.61 | 1.06 | Inf | 0.58 | 1.00 |
| K - NK | Two-lipped | -1.47 | 0.90 | Inf | -1.63 | 0.73 |
| K - NP | Two-lipped | 0.49 | 0.91 | Inf | 0.54 | 1.00 |
| K - NPK | Two-lipped | 0.24 | 0.77 | Inf | 0.31 | 1.00 |
| K - P | Two-lipped | -0.75 | 0.68 | Inf | -1.11 | 0.95 |
| K - PK | Two-lipped | -0.62 | 0.70 | Inf | -0.88 | 0.99 |
| N - NK | Two-lipped | -2.08 | 1.20 | Inf | -1.74 | 0.66 |
| N - NP | Two-lipped | -0.12 | 1.20 | Inf | -0.10 | 1.00 |
| N - NPK | Two-lipped | -0.38 | 1.10 | Inf | -0.34 | 1.00 |
| N - P | Two-lipped | -1.37 | 1.04 | Inf | -1.31 | 0.89 |
| N - PK | Two-lipped | -1.23 | 1.06 | Inf | -1.17 | 0.94 |
| NK - NP | Two-lipped | 1.96 | 1.07 | Inf | 1.84 | 0.59 |
| NK - NPK | Two-lipped | 1.71 | 0.95 | Inf | 1.79 | 0.63 |
| NK - P | Two-lipped | 0.72 | 0.88 | Inf | 0.81 | 0.99 |
| NK - PK | Two-lipped | 0.85 | 0.90 | Inf | 0.95 | 0.98 |
| NP - NPK | Two-lipped | -0.25 | 0.96 | Inf | -0.26 | 1.00 |
| NP - P | Two-lipped | -1.25 | 0.89 | Inf | -1.40 | 0.86 |
| NP - PK | Two-lipped | -1.11 | 0.91 | Inf | -1.22 | 0.93 |
| NPK - P | Two-lipped | -0.99 | 0.75 | Inf | -1.33 | 0.89 |
| NPK - PK | Two-lipped | -0.86 | 0.77 | Inf | -1.12 | 0.95 |
| P - PK | Two-lipped | 0.14 | 0.68 | Inf | 0.20 | 1.00 |
